# l- to d-Amino Acid Substitution in the Immunodominant LCMV-Derived Epitope gp33 Highlights the Sensitivity of the TCR Recognition Mechanism for the MHC/Peptide Structure and Dynamics

**DOI:** 10.1021/acsomega.1c06964

**Published:** 2022-03-07

**Authors:** Federico Ballabio, Luca Broggini, Cristina Paissoni, Xiao Han, Kaliroi Peqini, Benedetta Maria Sala, Renhua Sun, Tatyana Sandalova, Alberto Barbiroli, Adnane Achour, Sara Pellegrino, Stefano Ricagno, Carlo Camilloni

**Affiliations:** † Dipartimento di Bioscienze, Università degli Studi di Milano, Milano 20133, Italy; ‡ Institute of Molecular and Translational Cardiology, IRCCS Policlinico San Donato, San Donato Milanese 20097, Italy; § Science for Life Laboratory, Department of Medicine, Karolinska Institute, & Division of Infectious Diseases, Karolinska University Hospital, Stockholm 14186, Sweden; ∥ DISFARM, Dipartimento di Scienze Farmaceutiche, Sezione Chimica Generale e Organica, Università degli Studi di Milano, Milano 20122, Italy; ⊥ Dipartimento di Scienze per gli Alimenti, la Nutrizione e l’Ambiente, Università degli Studi di Milano, Milano 20122, Italy

## Abstract

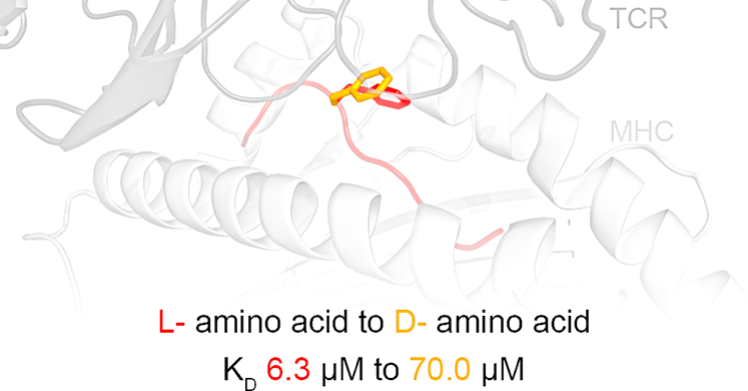

Presentation of pathogen-derived epitopes by major histocompatibility complex I (MHC-I) can lead to the activation and expansion of specific CD8^+^ T cell clones, eventually resulting in the destruction of infected target cells. Altered peptide ligands (APLs), designed to elicit immunogenicity toward a wild-type peptide, may affect the overall stability of MHC-I/peptide (pMHC) complexes and modulate the recognition by T cell receptors (TCR). Previous works have demonstrated that proline substitution at position 3 (p3P) of different MHC-restricted epitopes, including the immunodominant LCMV-derived epitope gp33 and escape variants, may be an effective design strategy to increase epitope immunogenicity. These studies hypothesized that the p3P substitution increases peptide rigidity, facilitating TCR binding. Here, molecular dynamics simulations indicate that the p3P modification rigidifies the APLs in solution predisposing them for the MHC-I loading as well as once bound to H-2D^b^, predisposing them for TCR binding. Our results also indicate that peptide position 6, key for interaction of H-2D^b^/gp33 with the TCR P14, takes a suboptimal conformation before as well as after binding to the TCR. Analyses of H-2D^b^ in complex with APLs, in which position 6 was subjected to an l- to d-amino acid modification, revealed small conformational changes and comparable pMHC thermal stability. However, the l- to d-modification reduced significantly the binding to P14 even in the presence of the p3P modification. Our combined data highlight the sensitivity of the TCR for the conformational dynamics of pMHC and provide further tools to dissect and modulate TCR binding and immunogenicity via APLs.

## Introduction

The inner health status of most cells is mirrored at their surfaces by the immunopeptidome, a large ensemble of processed peptides, which are presented to the immune system by class I and class II major histocompatibility complex molecules (MHC-I and MHC-II, respectively).^1,2^ The interface surface that results from the combination of each peptide and MHC-I is key for recognition by the T cell receptor (TCR) on CD8^+^ T cells. Thus, presentation of a pathogen-derived epitope by an MHC-I molecule will most often result in the activation and expansion of specific CD8^+^ T cell clones, which ultimately should lead to the destruction of infected target cells by activated cytotoxic CD8^+^ T lymphocytes (CTLs).^3−5^ Furthermore, recent developments in mass spectrometry have significantly enhanced our capacity to unambiguously identify MHC-I- and MHC-II-restricted tumor-associated antigens (TAAs) and have thus opened new avenues for cancer treatment.^6−8^

Many studies have investigated the role of the presented epitope in tuning stability and flexibility of MHC/peptide (pMHC) complexes and its relation to immunogenicity. X-ray crystallography, nuclear magnetic resonance (NMR) spectroscopy, fluorescence, and molecular dynamics (MD) simulation studies have indeed revealed that the presented peptides retain different degrees of flexibility within the cleft and can also populate multiple conformations.^9−20^ Importantly, specific modifications in altered peptide ligands (APLs) may have significant effects on the overall stability of pMHC complexes, recognition by TCRs, and immunogenicity.^21−24^ The modifications introduced in MHC-I-restricted APLs should not alter their conformations compared to their wild-type counterparts because the main intention is to elicit strong responses toward tumors and/or pathogens by increasing the capacity of modified more stable pMHCs to recruit and activate adequate T cell populations, eliciting immune responses toward previously ignored targets through molecular mimicry and T cell cross-reactivity.^25−28^

Achour and co-workers have previously demonstrated that vaccination with H-2D^b^-restricted APLs, in which peptide position 3 was modified to a proline (p3P), increased significantly immune responses toward targets presenting tumor-associated epitopes.^23,25,26,29−31^ Indeed, the p3P modification in the H-2D^b^-restricted melanoma-associated TAA gp100_25-33_ and the TEIPP-neoantigen Trh4 increased binding affinity to their cognate TCRs pMel and LnB5, respectively, resulting in significantly stronger in vivo and in vitro immune responses by endogenous CD8^+^ T cell populations toward cancer targets. However, while the p3P-modified APL gp100_25-33_(p3P) significantly increased the stability of the pMHC complex,^26^ the high immunogenicity following vaccination with Trh4-p3P was unrelated to complex stability because the stability of H-2D^b^/Trh4-p3P was significantly reduced compared to that of H-2D^b^/Trh4.^23^ This confounding result, coupled to X-ray crystallography-based comparative analyses, led therefore Achour and co-workers to hypothesize that the p3P modification induces a rigidification of the APLs, facilitating TCR recognition. More recently, Achour and co-workers also addressed whether vaccination with p3P-modified APLs would enable the tuning of endogenous CD8^+^ T cell recognition toward a viral immune escape variant. It is well established that infection of C57/Bl6 mice with lymphocytic choriomeningitis virus (LCMV) induces robust CTL responses toward the immunodominant H-2D^b^-restricted epitope gp33 (KAVYNFATM).^32−34^ Upon CTL pressure, a limited number of mutations in gp33 emerge, with consistent patterns, allowing for viral escape from CD8^+^ T-cell recognition.^35^ One of the main naturally occurring mutations that allows efficient escape by LCMV is the p4F substitution (Y4F, KAVFNFATM), which abrogates endogenous CD8^+^ T cell recognition as well as recognition by the H-2D^b^/gp33-specific TCR P14. It was then demonstrated that peptide vaccination with the p3P-modified version of the escape variant V3P_Y4F (KAPFNFATM) efficiently restores recognition of infected cells presenting Y4F by endogenous CD8^+^ T cells in LCMV-infected mice.^25^ To assess the molecular bases underlying TCR recognition of V3P_Y4F compared to Y4F, the crystal structures of each pMHC were compared in ref. 25, before and after TCR P14 binding, revealing that (i) P14 binds nearly identically to all pMHC complexes and (ii) the conformations of peptide residues p1K and p6F as well as H-2D^b^ residues R62, E163, and H155 are affected by the p3P modification, seemingly predisposing pMHC complexes for TCR recognition. Furthermore, circular dichroism, surface plasmon resonance (SPR), and isothermal titration calorimetry results also demonstrated that each p3P-APL increased pMHC complex stability and facilitated P14 recognition through reduced entropy costs.^25^ The analysis of B-values in the crystal structures indicated that the p3P modification rigidified APLs compared to their wild-type counterparts, thus that the molecular bases for the observed affinity and functional effects of the p3P were likely related to a decrease in dynamics in the targeted pMHC complexes.^25^

**Figure 1:**
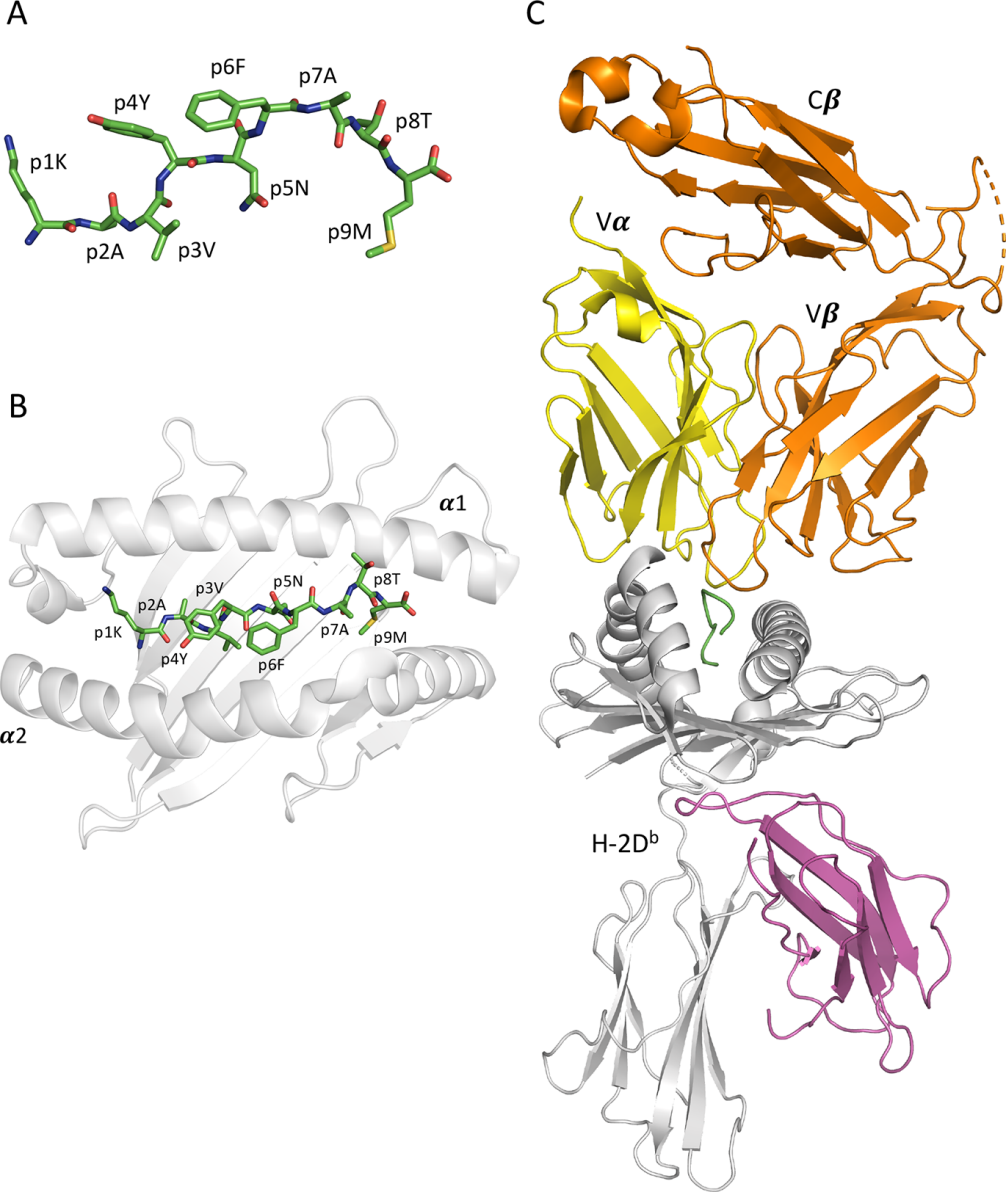
3d structures of the peptide gp33 in solution, bound to H-2D^b^ and in ternary complex with the TCR P14. This MD simulation study focuses on three different structural conditions for gp33 and its APL variants, exemplified by (A) gp33 (KAVYNFATM) alone in solution (carbon, nitrogen, oxygen, and sulfur atoms are colored green, blue, red, and yellow, respectively); (B) gp33 in complex with the peptide-binding cleft of H-2D^b^ composed of the heavy chain domains α1 and α2 (gp33 is colored as described above and the H-2D^b^ heavy chain backbone is white); and (C) H-2D^b^/gp33 in complex with the T-cell receptor P14 (the H-2D^b^ heavy chain is in white, the β_2_-microglobulin subunit in purple, the peptide in green, and the two TCR domains α and β are in yellow and orange, respectively).

Here, we compared using MD simulations the conformational dynamics of gp33 and of the p3P APL (V3P, KAPYNFATM), free in solution, bound to MHC (H-2D^b^/pep), and in complex with the TCR (P14/H-2D^b^/pep) (Figure 1). Our results indicate that the p3P modification rigidifies the APL both in solution as well as in pMHC, providing an explanation to its functionally observed higher capacity to bind to H-2D^b^ and to stabilize more efficiently the pMHC complexes. The overall rigidification of the APL also predisposes it for TCR recognition. Ramachandran analyses revealed that peptide position 6, key for adequate interactions with the TCR, takes an unfavorable conformation in both the H-2D^b^/pep and P14/H-2D^b^/pep complexes. Thereafter, we designed in silico a l- to d-substitution meant to further probe this position. Two new modified peptides (F6f and V3P_F6f) were experimentally characterized showing small structural differences and a significantly reduced binding by the H-2D^b^/gp33-specific TCR P14.

## Results

### p3P Substitution Rigidifies the Peptide, Increasing Its Predisposition for pMHC Loading and TCR Binding

**Figure 2:**
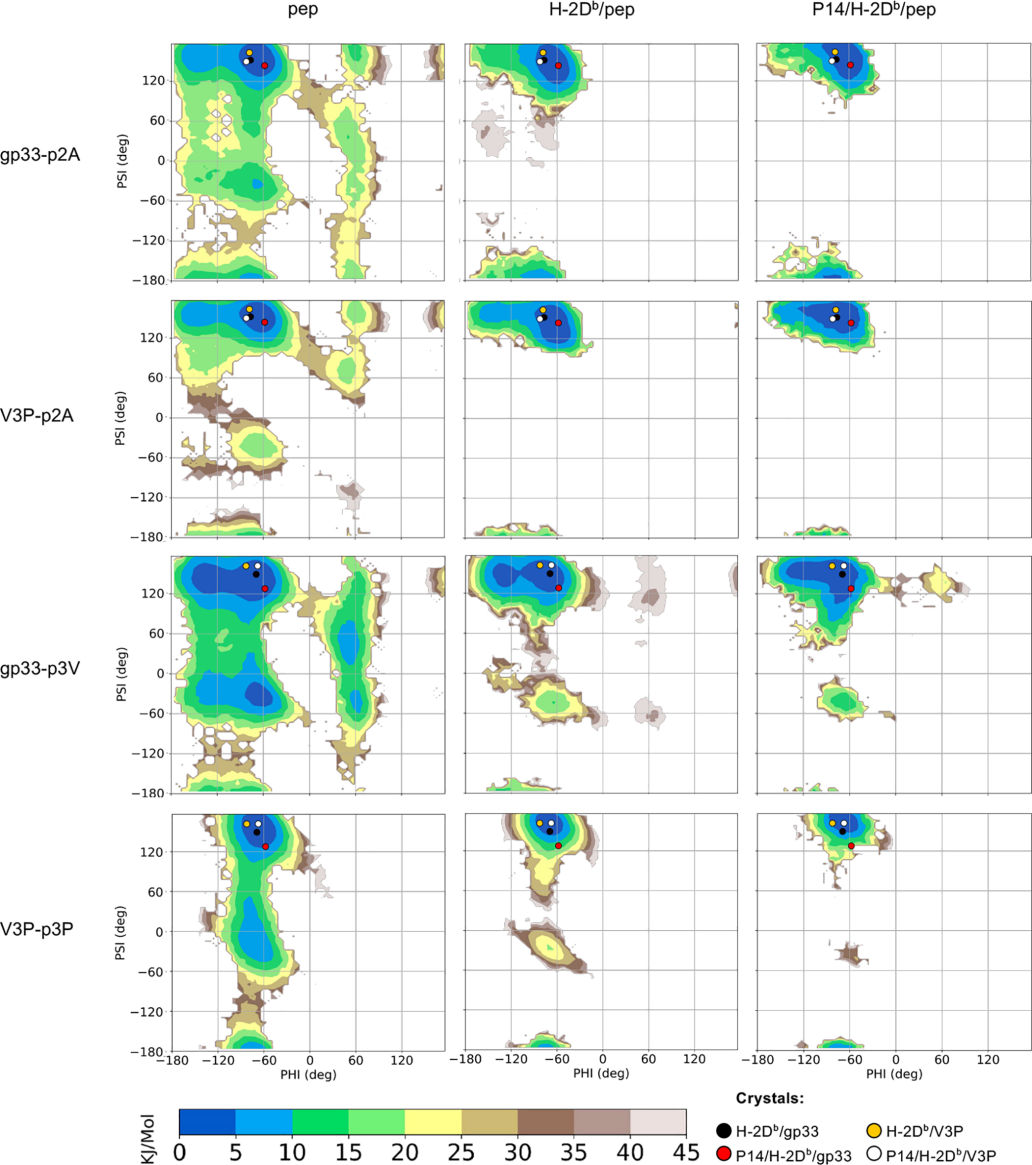
Ramachandran-like free energy surfaces indicate that the p3P modification reduces significantly the available conformational space for peptide residue p2A both in solution and when bound to H-2D^b^. The graphs present 2d free energies surfaces, as a function of the phi and psi dihedral angles, for peptide residue p2A in gp33 (top panel) and in V3P (second panel), as well as for residue p3V in gp33 (third panel) and p3P in V3P (bottom panel). The free energies are reported for both gp33 and the V3P peptides, for the simulations of the peptide (pep) alone (left), H-2D^b^/pep (middle), and P14/H-2D^b^/pep (right). In all graphs, the coordinates of the crystallographic structures for each corresponding residue are plotted as colored dots.

To better assess the effects of the p3P modification on peptide rigidity, we compared, using parallel-bias metadynamics simulations,^36^ the conformational freedom of gp33 (KAVYNFATM) and the APL V3P (KAPYNFATM) when (i) alone in solution, (ii) bound to H-2D^b^ (H-2D^b^/pep), and (iii) forming a ternary complex with the TCR P14 (P14/H-2D^b^/pep) (Figure 1). The conformational spaces of the peptides gp33 and V3P in solution, H-2D^b^/pep, or P14/H-2D^b^/pep complexes were analyzed in terms of the propensity of each peptide residue to visit different configurations as identified using the Ramachandran plot. Free energy surfaces (FES) for both gp33 and V3P are presented in Figure 2 as a function of the phi and psi backbone angles of residues p2A and p3V/p3P. The FES indicate that, in solution, peptide residues 2 and 3 in both gp33 and V3P can explore all the accessible Ramachandran space (Figure 2). However, as expected considering the restricted Ramachandran profile of proline residues, the p3P modification reduced the conformational freedom of these two residues in V3P in solution compared to gp33 (Figure 2). A single minimum is then selected following binding of either gp33 or V3P to H-2D^b^ and is not further modified by binding to the TCR P14. Considering the whole peptide, a principal component analysis of the simulations (Figure S1) also showed that the MHC loading restricts the peptide into an extended conformation.

**Figure 3:**
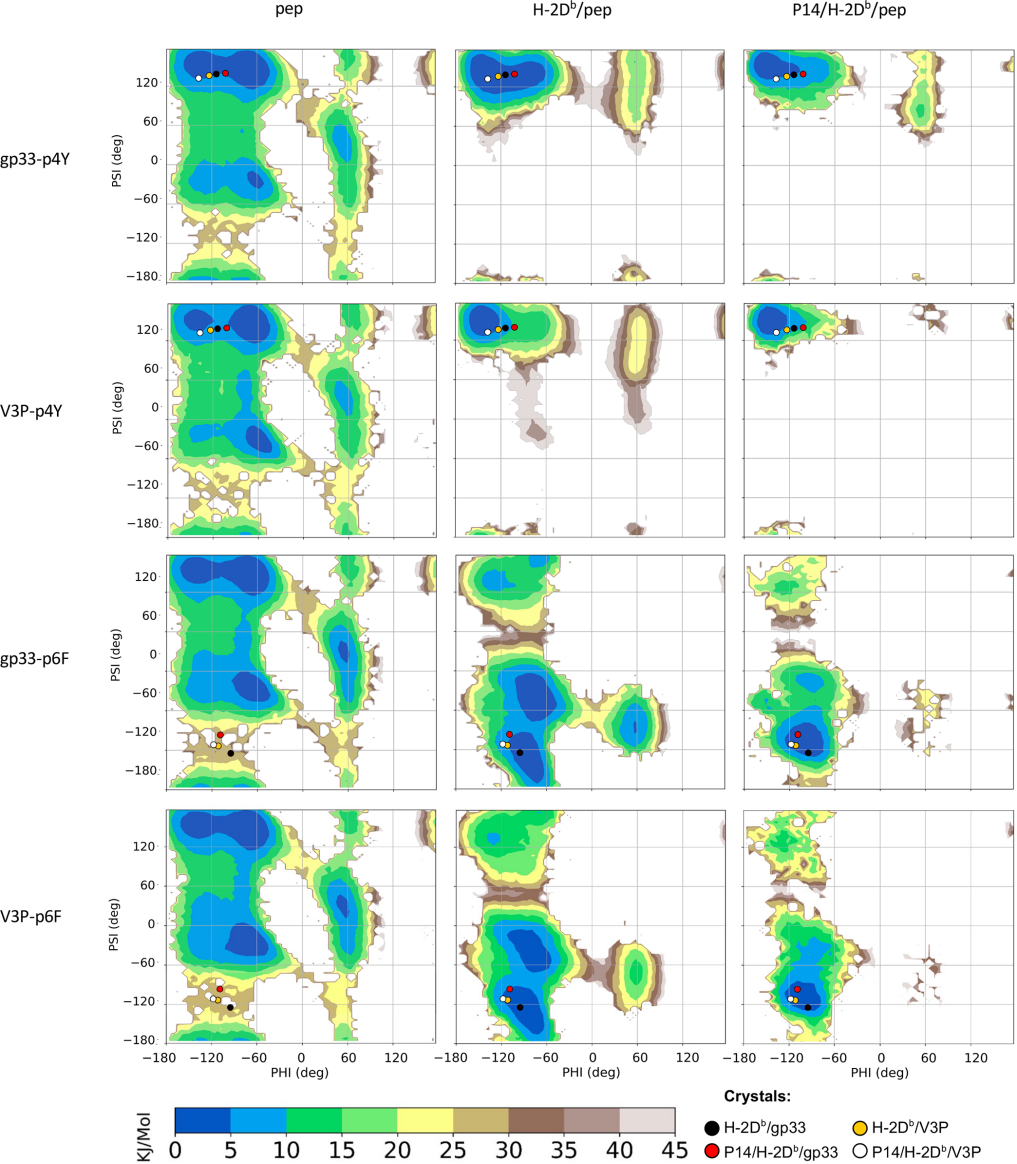
Ramachandran-like free energy surfaces for the TCR-interacting peptide residue p4Y and p6F indicate that the p3P modification slightly reduces the available conformational space of p4Y and not that of p6F. The 2d-free energies surfaces are presented, as a function of the phi and psi dihedral angles, for peptide residues p4Y (top panels) and p6F (bottom panels). The free energies are reported for both gp33 and the V3P peptides, for the simulations of peptides alone (left), H-2D^b^/pep (middle), and P14/H-2D^b^/pep complexes (right). In all the plots, the coordinates of residues p4Y and p6F in the four previously determined crystal structures are plotted as colored dots.

The p3P modification in V3P did not reduce the conformational space of any other peptide residue in solution (Figures 3 and S2), instead, although the overall effect is mild, the p3P modification reduced the conformational space for the peptide residue p4Y when bound to H-2D^b^. This latter effect can be observed both for the p4Y backbone, which displays a narrower minimum as compared to gp33 (Figure 3), and for the p4Y side chain, as monitored by means of the χ_1_ and χ_2_ dihedral angles (Figure S3). While these effects are weak, they appear to be significant as the presented free energies are well converged (see Figures S4 and S5).

**Figure 4:**
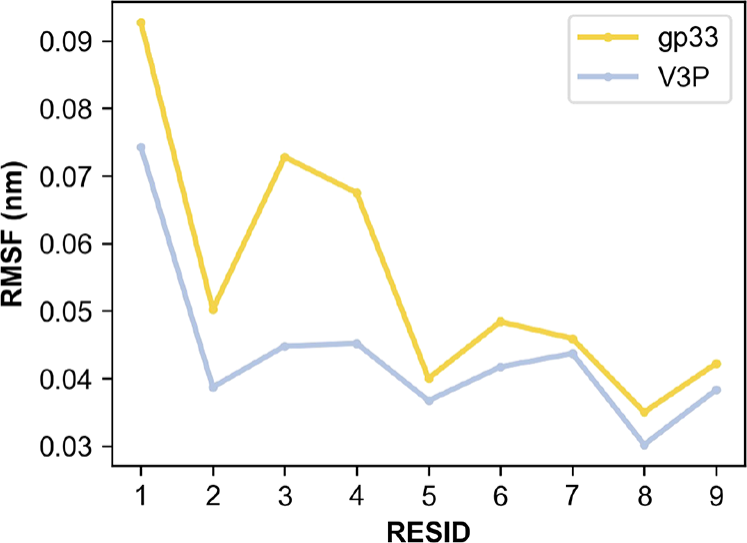
p3P modification results in a rigidification of the APL within the H-2D^b^ cleft. Per-residue backbone RMSFs following MD simulations of the peptides gp33 and V3P when bound to H-2D^b^ indicate reductions of fluctuations at the four N-termini peptide positions. It should be noted that the main anchor positions defining H-2D^b^-restriction are at positions 5 and 9.

Root mean square fluctuation (RMSF) analysis for the peptides in the H-2D^b^/gp33 and the H-2D^b^/V3P complexes indicates that the p3P substitution increases the rigidity of the peptide with a marked effect on residues p3 and p4 (Figure 4).

Altogether, these results indicate that the p3P substitution restricts mostly the conformational space of residues p2-p3 in the modified peptides already in solution, thus suggesting that loading V3P into the peptide-binding cleft of H-2D^b^ may cost less entropy compared to gp33, resulting in a more stable H-2D^b^/pep complex.^37,38^ This restriction is further extended to p4 in H-2D^b^/pep, supporting the idea that the p3P modification can favor the preorganization of the peptide for binding to the TCR confirming the previous hypothesis by Achour and co-workers.^23,25,26,30^

Interestingly, our MD simulation analyses indicate that peptide residue p6F displays a significantly more dynamic behavior in both gp33 and V3P, even when loaded into the peptide-binding cleft of H-2D^b^ (Figure 3). Indeed, loading of either gp33 or V3P into H-2D^b^ results in a complete reshaping of the FES for p6F, with new minima displayed in regions that are generally not favorable for the backbone (Figure 3). Furthermore, peptide residue 6 seems to also have a larger conformational freedom with respect to other peptide positions when bound to the TCR (Figures 3 and S2).

### In Silico Design of an l- to d-Substitution at Peptide Position 6 is Compatible with H-2D^b^ Loading and P14 TCR Binding

The results presented above indicate that binding of gp33 to H-2D^b^ is optimal for all peptide positions but for residue p6F that is forced to populate a region of the Ramachandran plot that is only marginally populated by the peptide in solution. The conformational strain of this specific peptide residue is also reflected by the larger conformational freedom of p6F in the P14/H-2D^b^/pep complex. Inspection of the P14/H-2D^b^/gp33 complex crystal structure (PDB code 5TJE) indicated that the source of this strain could be the presence of two interacting tryptophan heavy chain residues (W73 and W147). These two residues create a “bulge” in the MHC binding pocket forcing the bound peptides to adopt an unfavorable conformation around residues p6 and p7 (Figure S6).^39,40^

**Figure 5:**
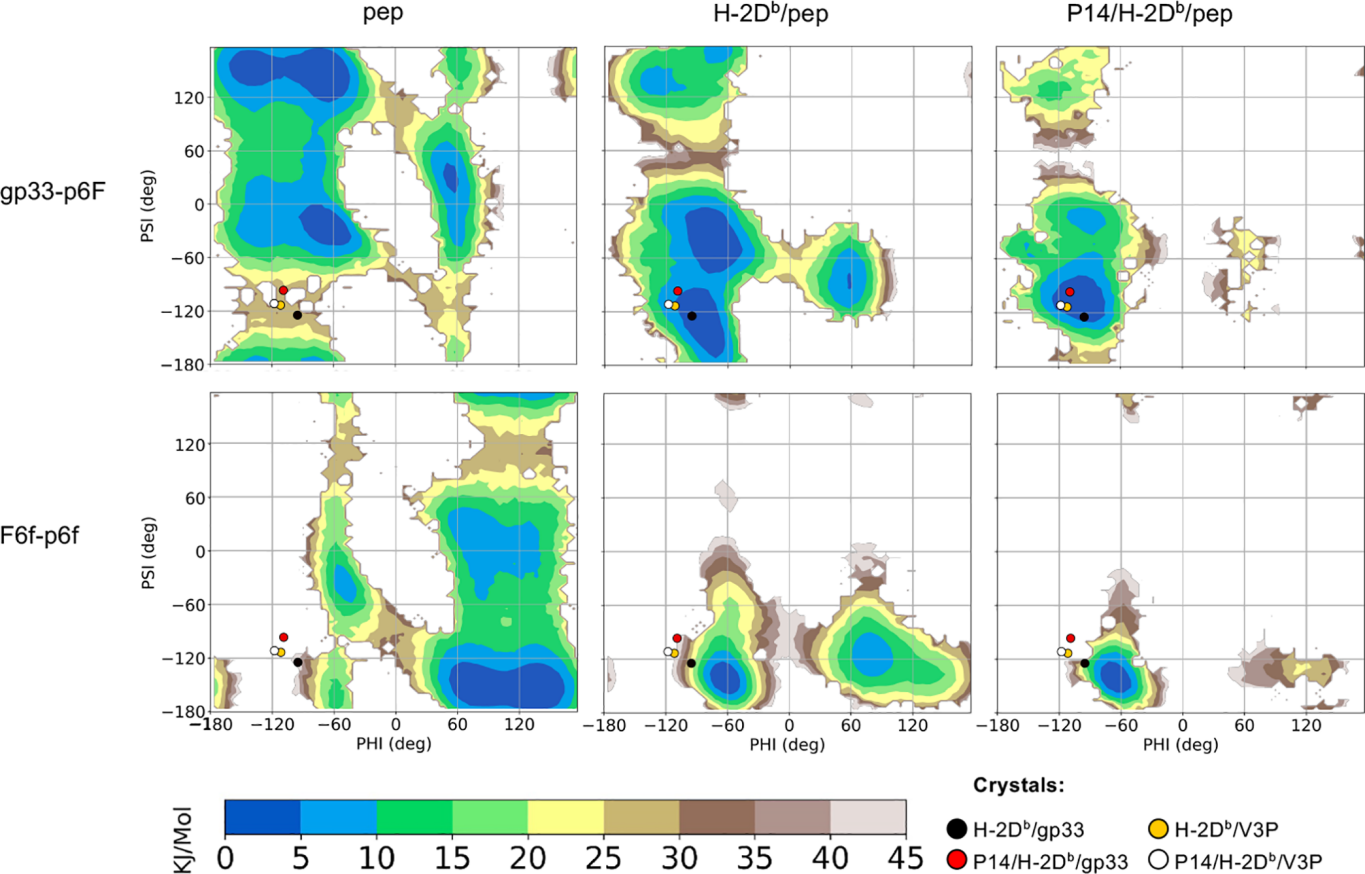
Ramachandran-like free energy surfaces for residues p6F and p6f in gp33 and the designed F6f peptide. The 2d-free energy surfaces, as a function of the phi and psi dihedral angles of residues p6F and p6f, are presented for gp33 (top panel) and F6f (bottom panel), respectively, for the simulations of the peptides alone (left), H-2D^b^/pep complexes (middle), and P14/H-2D^b^/pep complexes (right). In all the plots, the coordinates of the corresponding residues in the crystal structures comprised within this study are plotted as colored dots.

These observations, and especially the fact that the conformational strain at peptide position 6 is present in a large majority of structurally determined H-2D^b^-restricted epitopes^39^ (Figure S7) raised our curiosity. To better understand the possible importance of the conformation of this specific residue for pMHC stability and for TCR recognition, we aimed at reducing the strain of the backbone without changing the side chain of residue p6F. We therefore designed an l- to d-phenylalanine peptide variant for gp33, which resulted in the new APL F6f (KAVYN**f**ATM, where the lower-case f indicates d-phenylalanine). In Figure 5, we compare the FES for peptide residue 6 in gp33 and in the newly designed F6f, loaded into H-2D^b^ and when bound to the TCR P14. Our results indicate that the peptide F6f in solution still cannot explore the conformations observed for position 6 in the crystal structures of H-2D^b^ in complex with gp33 or V3P. At the same time our simulations in the context of H-2D^b^/pep and P14/H-2D^b^/pep complexes suggest that the l- to d-modified peptide F6f is compatible with the H-2D^b^ loading and TCR P14 binding, even if with a slightly different geometry (Figure 5). Of note, the l- to d-modification decreases the conformational freedom, that is, the number of free energy minima, of peptide position 6 in molecular models of both H-2D^b^/F6f and P14/H-2D^b^/F6f complexes. Furthermore, comparative RMSF analysis for H-2D^b^/F6f and H-2D^b^/gp33 does not indicate any significant difference between the two complexes (Figure S8), suggesting a minor rigidification effect in F6f compared to the stronger effects following the p3P modification in V3P.

### l- to d-Modification Reduces Significantly TCR Recognition

**Figure 6:**
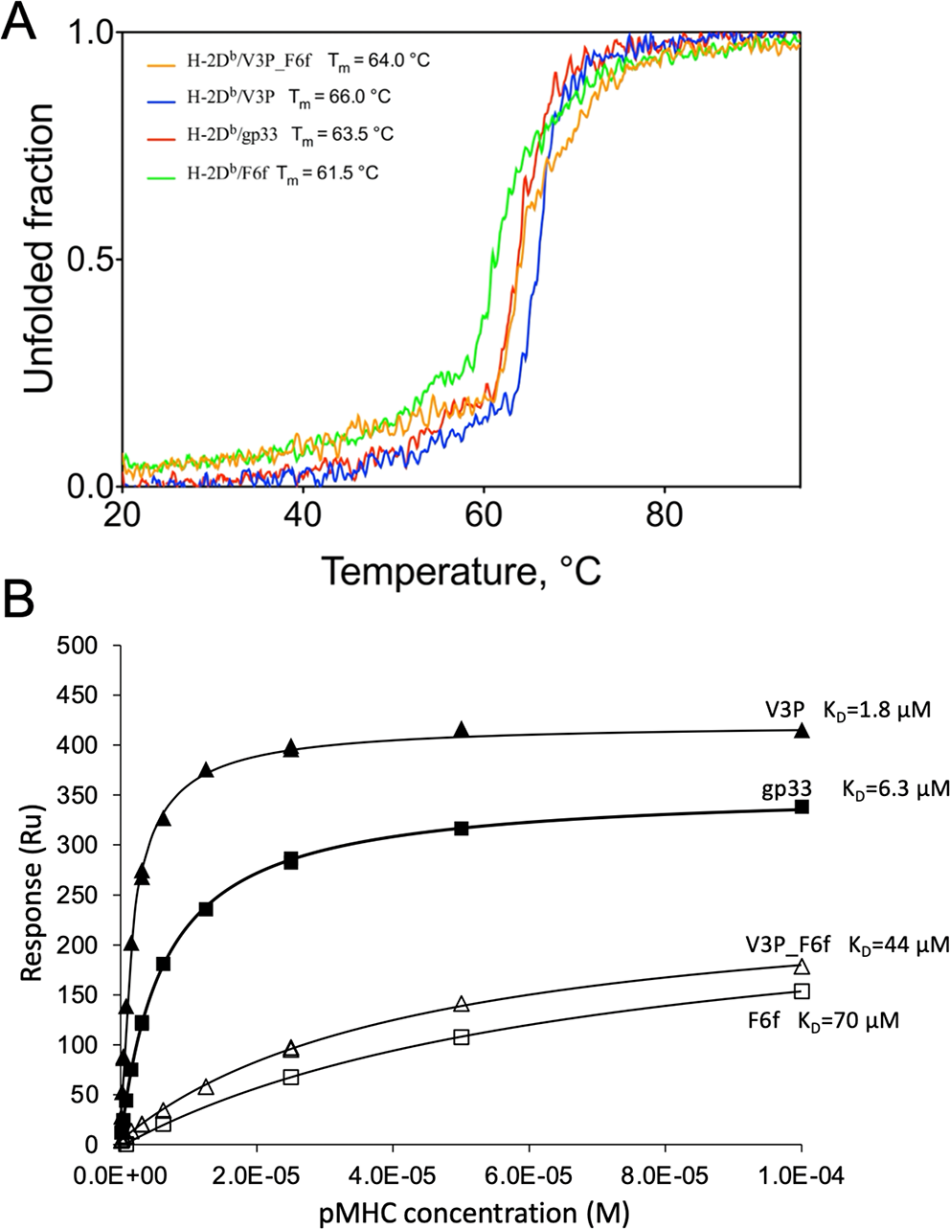
l- to d-substitution significantly affects recognition by the TCR P14. (A) Circular dichroism unfolding profiles demonstrate that the l- to d-modification of peptide residue p6f reduces slightly the overall stability of the H-2D^b^/gp33 and H-2D^b^/V3P complexes. Thermal denaturation unfolding curves of the pMHC-I complexes included within the present study were monitored in the far-UV region by circular dichroism. Melting temperatures (*T*_m_) corresponding to 50% protein denaturation are indicated. (B) l- to d-substitution significantly reduces the binding affinity of the TCR P14. The binding affinity of the soluble TCR P14 to each pMHC was measured using SPR. *K*_D_ values are indicated.

To evaluate the effect of the l- to d-modification in p6 on the stability of the H-2D^b^/F6f or the H-2D^b^/V3P_F6f (which combined p3P and p6f modifications) complexes, we made use of circular dichroism and performed thermal unfolding ramps in the far-UV region (Figure 6A). All pMHC complexes displayed a high degree of cooperativity during thermal unfolding. The *T*_m_ values derived from the unfolding curves indicate a slight decrease in stability for H-2D^b^ presenting l- to d-modified peptides compared to their unmodified counterparts (Figure 6A).

Most importantly, SPR analyses revealed a 10-fold lower binding affinity of soluble P14 TCR to H-2D^b^/F6f compared to H-2D^b^/gp33 (70 and 6.3 μM, respectively). A similar significant decrease in affinity was also observed for binding of P14 to H-2D^b^/V3P_F6f compared to H-2D^b^/V3P (44 and 1.8 μM, respectively) (Figure 6B). These results highlight the importance of the specific configuration, although strained, assumed by position 6 for adequate recognition by the TCR P14.

### l- to d-Substitution in F6f Modifies Binding Cleft Geometry

**Figure 7:**
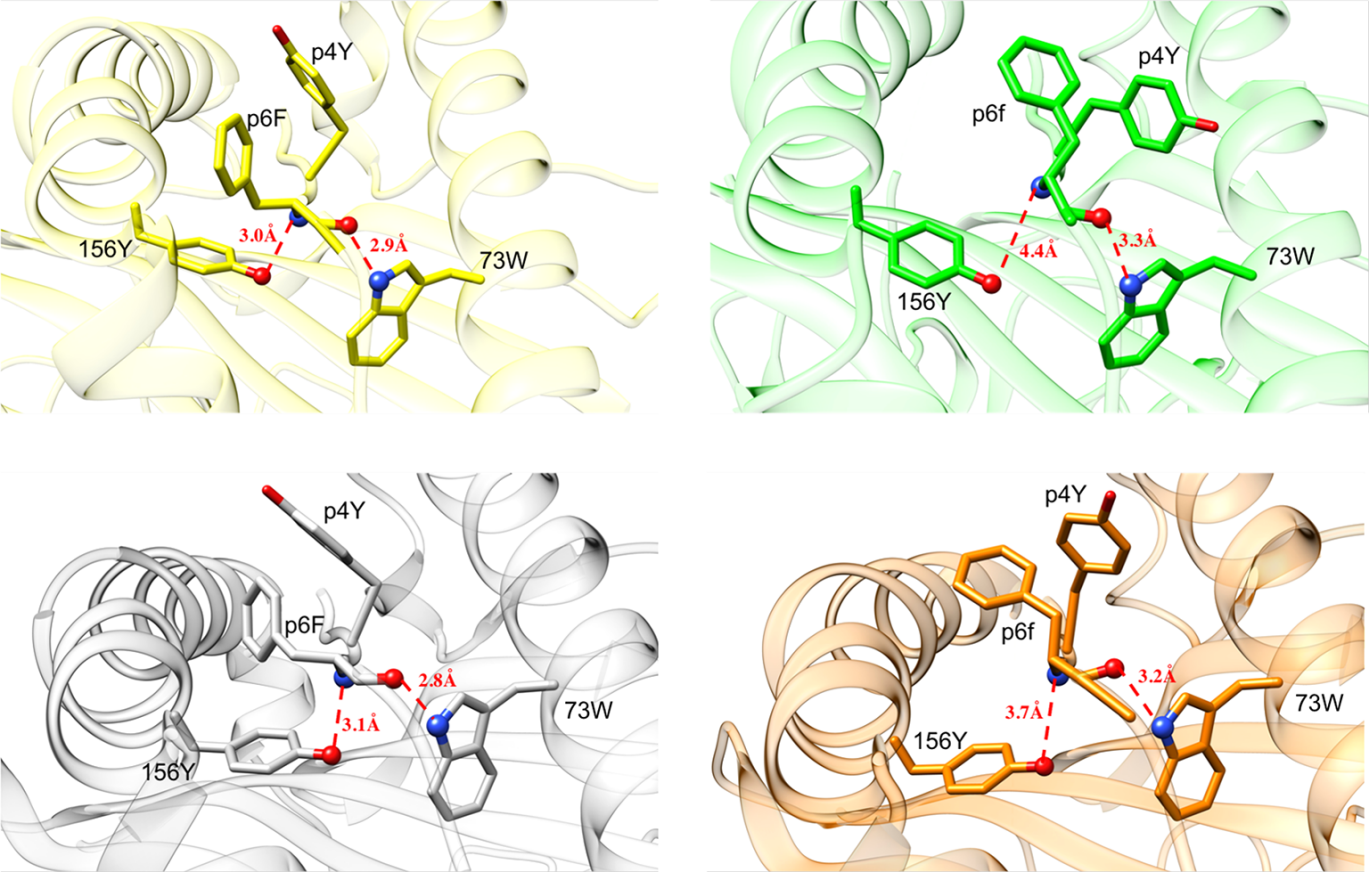
Spatial orientation of p6f induces changes in the local geometry within the pMHC-binding cleft. Side view of H-2D^b^/gp33 (yellow), H-2D^b^/F6f (green), H-2D^b^/V3P (grey), and H-2D^b^/V3P_F6f (orange) binding clefts. H-2D^b^ Y156:OH–p6F:NH and H-2D^b^ W73:NH–p6F:CO atomic distances are highlighted. The abovementioned distances are increased in the l- to d-substituted pMHC complexes compared to their counterparts. The introduction of p6f in the peptide backbone causes a less tight hydrogen bond network within the binding cleft, with an increase of hydrogen bond distances.

**Figure 8:**
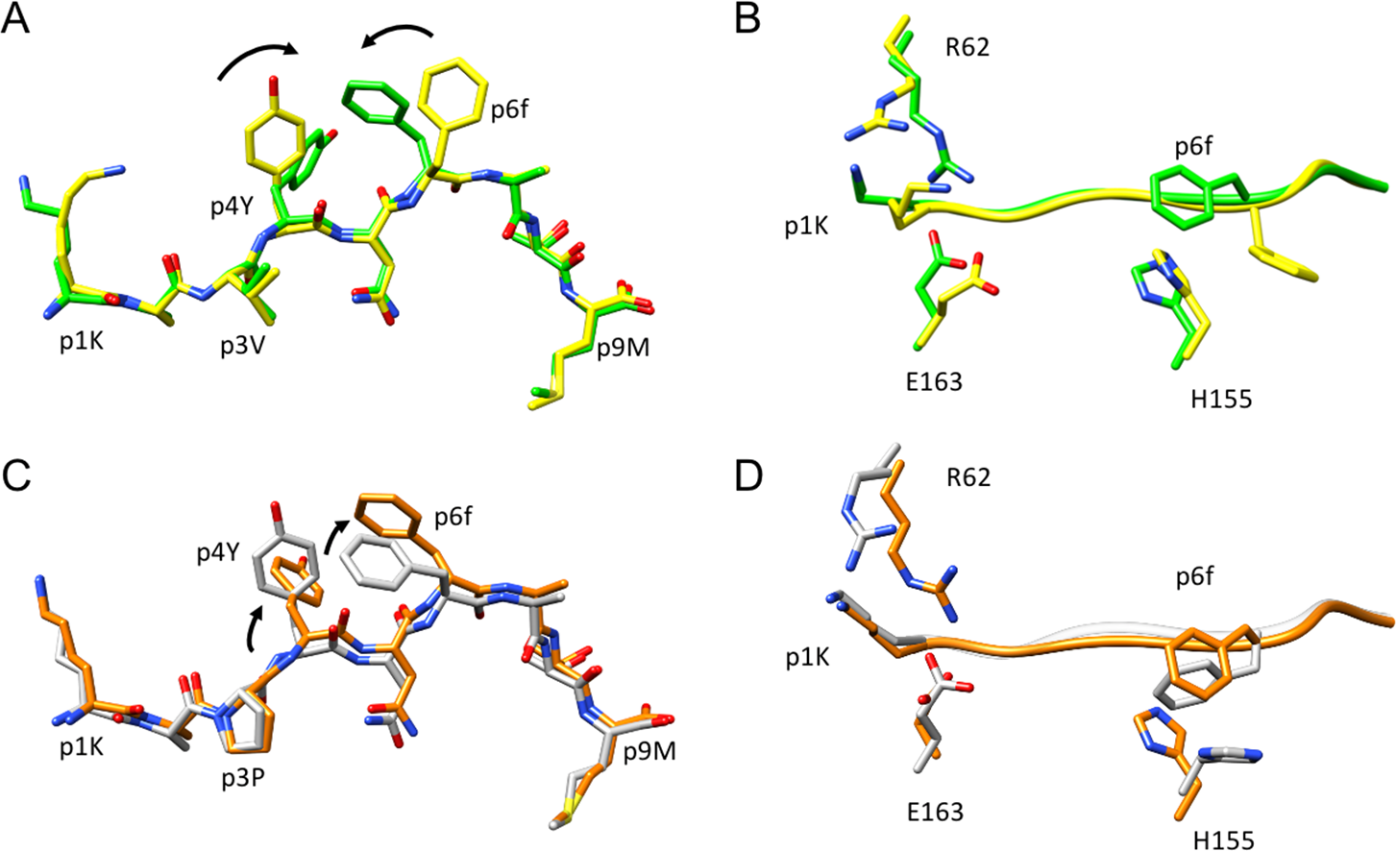
The l- to d-substitution in F6f and V3P_F6f introduces modifications on peptide and H-2D^b^ key residue orientation. (A,C) Comparison of the crystal structures of H-2D^b^/F6f and H-2D^b^/gp33 reveal that the backbone of peptides F6f (green) and gp33 (yellow) takes highly similar conformations. The side chains of residues p1K, p4Y, and p6f take different conformations compared to the same residues in gp33. Comparison of the crystal structures of H-2D^b^/V3P_F6f and H-2D^b^/V3P reveals also similar conformations, with a slight movement upwards of the p4-p6 region. In both cases, the conformation of the side chain of p4Y is affected by the introduction of the l- to d-substitution at peptide position 6. (B,D) l- to d-modification results in a different conformation of p6f compared to p6F, potentially hindering the H155 clockwise movement that should occur following binding of the TCR P14.

To assess the molecular bases underlying the effects of the l- to d-substitution at peptide position 6 on TCR affinity, we determined the crystal structures of H-2D^b^/F6f and H-2D^b^/V3P_F6f to be 2.6 and 2.4 Å resolution, respectively (Table 1 and Figures 7 and 8). The crystal structures of the H-2D^b^/F6f and H-2D^b^/V3P_F6f complexes were then compared with the previously determined crystal structures of H-2D^b^/gp33 and H-2D^b^/V3P as well as with the corresponding MD simulation results. Starting from the backbone of peptide position 6, we observe that the phi and psi dihedral angles calculated from the determined crystal structures fall in the main minimum predicted by the corresponding MD simulations of H-2D^b^/F6f and P14/H-2D^b^/F6f. This minimum is slightly shifted with respect to the corresponding minimum in MD simulations performed on H-2D^b^/gp33, thus resulting in a slightly different conformation of peptide F6f compared to gp33 (Figure S9). These differences can be further highlighted in the crystal structures measuring the distance between the NH of H-2D^b^ residue W73 and the carboxyl group of p6 as well as that between the amide group of p6 and the hydroxyl group of the H-2D^b^ residue Y156. In H-2D^b^/F6f and H-2D^b^/V3P_F6f, both distances increase in comparison to the corresponding structures with the l-amino acid (Figure 7).

**Table 1:** Data Collection and Refinement Statistics for the Crystal Structures of the H-2D^b^/V3P_F6f and H-2D^b^/gp33-F6f complexes^a^

data set	H-2D^b^/V3P-_F6f	H-2D^b^/F6f
PDB code	7P0A	7P0T
Data Collection		
space group	C2	C2
cell dimensions		
*a*, *b*, *c* (Å)	120.6, 126.0, 92.9	120.5, 124.6, 92.9
α, β, γ, (deg)	90.0, 126.6, 90.0	90.0, 126.8, 90.0
wavelength (Å)	1.000	1.000
Resolution range (Å)		
upper limit along reciprocal axes*	2.38, 2.98, 2.61 (2.68–2.43)	2.56, 3.44, 2.79 (2.89–2.60)
lower limit	76.76	48.2
^#^*R*_pim_	0.045 (0.416)	0.028 (0.309)
+CC_1/2_	0.994 (0.591)	0.998 (0.773)
⟨*Ι*/σ(*Ι*)⟩	10.5 (2.0)	16.1 (2.7)
redundancy	6.5 (7.0)	6.5 (6.7)
Completeness (%)		
spherical	72.1 (16.7)	65.2 (13.6)
ellipsoidal	93.2 (60.8)	91.5 (59.1)
Refinement		
resolution (Å)	76.76–2.43 (2.68–2.43)	48.2–2.60 (2.89–2.60)
number of reflections	30298 (1782)	22227 (1309)
*R*_work_/*R*_free_	0.211/0.256	0.197/0.244
Number of Molecules		
copies in the AU	2	2
protein residues	695	689
water molecules	118	80
average *B* factors (Å^2^)	84.2	82.5
Rmsd		
bond lengths (Å)	0.003	0.005
bond angles (deg)	0.68	0.77
Ramachandran Plot Statistics		
most favored region	673 (96.8%)	668 (97.0%)
allowed region	20 (2.9%)	19 (2.7%)
outliers	2 (0.3%)	2 (0.3%)

a Values in parentheses are for the highest resolution shell. *R*_merge_ = Σ_*hkl*_Σ_*j*_*I*_*hkl*,*j*_ – ⟨*I*_hkl_⟩/Σ_*hkl*_Σ_*j*_*I*_*hkl*,*j*_, where *I* is the observed intensity and <I> is the average intensity. *R*_work_ = Σ_*hkl*_*F*_o_ – *F*_*c*_/Σ_*hkl*_*F*_o_ for all data except 5–10%, which were used for the *R*_free_ calculation.

As a result of the l- to d-symmetry, the side chain of p6f takes a different conformation in H-2D^b^/F6f compared to H-2D^b^/gp33 (Figure 8A). Interestingly, the aromatic ring of peptide residue p4Y, essential for recognition by the TCR P14, is profoundly affected by p6f and rotates clockwise from its conformation in gp33. The situation is very similar in H-2D^b^/V3P_F6f compared to H-2D^b^/V3P (Figure 8C). The different spatial conformation adopted by p4Y and p6f in H-2D^b^/F6f and H-2D^b^/V3P_F6f complexes is reflected also in the reorganization of H-2D^b^ residues involved in TCR binding, namely H155, R62, and E163 (Figure 8B, D). In particular, the rotation of p6f in H-2D^b^/V3P_F6f prevents the H-2D^b^ residue H155 to rotate counter clock wise and to assume a TCR-bound-like conformation, in contrast to that observed in H-2D^b^/V3P (Figure 8). Differences in the position of the H-2D^b^ residues R62 and E163 in both H-2D^b^/F6f and H-2D^b^/V3P_F6f are also observed. It should be noted, nonetheless, that in the MD simulations residues H155, R62, and E163 display similar dynamics for all the H-2D^b^/pep complexes, irrespectively of the loaded peptide (Figures S10–S12).

Altogether, the crystal structures of H-2D^b^/F6f and H-2D^b^/V3P_F6f revealed that the introduction of an l- to d-modification in peptide position 6 in gp33 does not compromise complex formation.

The geometrical perturbations within the binding cleft are compatible with the measured reduced thermal stability. The latter, possibly combined with the different spatial orientations of key heavy chain residues observed in the crystal structures, and with the differences in the conformational space populated by peptide residues 6 as observed in the simulations, may contribute to explain the reduced P14 affinity observed for both H-2D^b^/F6f and H-2D^b^/V3P_F6f.

## Discussion

MHC-I molecules are not rigid scaffolds. Instead, each pMHC combination seems to be characterized by unique properties related to the structure, stability, and dynamics of the complex. The loading of an MHC-I allele with different peptides will result not only in different surface properties but also in different dynamics of the pMHC complexes, including differences in local fluctuations as well as in the population of multiple conformational states. Eventually, these properties may possibly contribute to determine the affinity with which the TCR will bind the complex.^41^ Baker and co-workers have previously shown that different peptides can modulate differently the dynamics of pMHC also in regions far from the binding groove.^18^ They have also shown how an increase in the pMHC flexibility can lead to a loss of immunogenicity irrespectively of the pMHC stability.^21,42^ Achour and co-workers have also shown that an increase in the stability of the pMHC complex does not always correspond to an increased affinity, while the correlation is more likely associated with the increased rigidity of the peptide, for example, due to the substitution of peptide position 3 to a proline.^23,25,29,30,32^

Here, we investigated using MD simulations first to which extent the loading of gp33 or V3P into H-2D^b^ and the subsequent binding by the TCR P14 restrict the dynamics of the peptide. Each amino acid of the peptide in solution displays a backbone flexibility that allows it to explore the whole compatible Ramachandran space. Once the peptide is loaded into the MHC-I, the backbone conformational freedom is dramatically reduced but some conformational flexibility is still permitted. The residual conformational freedom is then only minimally perturbed by the formation of the complex with the TCR.

MD simulation analyses indicate that the role of the proline substitution at peptide position 3 is 2-fold. First, the proline reduces the accessible Ramachandran space for both positions 2 and 3 for the peptide in solution, and the observed available configurations are compatible with those observed following loading onto H-2D^b^. Once loaded, the p3P-modified peptide is also overall more rigid, which can facilitate binding of a TCR based on a reduced entropic cost, in agreement with previous ITC measures.^25^ We speculate here that the p3P rigidification may be important for more efficient selection and binding to MHC class I molecules by the peptide loading complex (PLC).^43,44^ Interestingly, a previous study in which a succession of crystal structures of the same MHC class I molecule was determined in complex with the peptide of different lengths and a fragment from the tapasin scoop loop revealed that the N-terminal part of the epitopes is loaded first before being tested for the affinity of the C-terminal to the F-pocket within the MHC peptide-binding cleft.^38^ Thus, the specific rigidification of the N-terminal part of the peptide could lead to more enhanced selection and binding efficiency. Furthermore, structural and NMR studies on the interactions of tapasin or TAPBPR with MHC-I molecules revealed that the peptide-binding groove is held by either of these chaperoning molecules in a more open conformation, especially at the F-pocket. However, distortions provoked by tapasin or TAPBPR may also extend all through the extent of the peptide-binding cleft, resulting in structural perturbations even in the A- and B-pockets, which theoretically promote the release of low-affinity peptides.^45,46^ These mechanisms have also been previously investigated and described at the atomic level via MD simulations, in which the role of MHC-binding groove dynamics was highlighted.^47,48^ If so, one could hypothesize that the loading of p3P-modified peptides such as V3P that is overall more rigid should take place at a significantly reduced entropy costs compared to their wild-type counterparts. Altogether, these results imply that the p3P modification results in a rigidification of the N-terminal segment of the peptide prior to binding to H-2D^b^, possibly simplifying selection and loading by the PLC. Indeed, this peptide rigidification could also prevail while waiting for a TCR to bind, possibly facilitating recognition.

Our analysis also revealed that H-2D^b^-restricted peptides, likely due to the presence of two heavy chain tryptophan residues, force peptide residue 6 to adopt a suboptimal backbone configuration as also previously observed in multiple crystal structures.^40^ This bulge, which is not present in, for example, H-2K^b^, has been previously suggested to be key to determine the pattern of buried and exposed peptide residues,^32,39^ as well as to increase the solvent accessibility of p6.^39^ This configuration is extremely unfavorable for the peptide in solution, indicating that some energy should be used for H-2D^b^/peptide complexes to be formed (Figure 5). This unusual configuration prompted us to try to design a modified peptide version that could better fit to the optimal Ramachandran values. We hypothesized that a l- to d-amino acid substitution in the peptide residue p6F, mirroring the corresponding l-amino acid, could significantly decrease the local strain, and allow us to address the possible advantage procured by this strained conformation for adequate TCR recognition. Of note, d-amino acids are not used in general to modify peptide antigens because they assume configurations that are sterically not compatible with their l-analogues.^49^ APLs, including d-amino acids as well as non-natural amino acids, have been proposed as a strategy to modulate immunogenicity while increasing the lifetime of the peptide.^50,51^d-amino acids have been mostly employed in the context of retroinverted peptides, while single substitutions have been found to produce diverse behaviors^49,52,53^

Our biochemical, biophysical, and structural characterization indicate that the l- to d-amino acid substitution at p6F in gp33 and V3P is well tolerated by H-2D^b^/peptide complexes, providing to our knowledge the first crystal structures of pMHC complexes including a d-amino acid. The resulting pMHC structure is essentially identical to the wild type and is still characterized by high thermal stability. However, the introduction of the l- to d-modification significantly reduced the binding affinity of the TCR P14 to H-2D^b^/pep complexes, even in the presence of the p3P modification. This indicates the high importance of the strained conformation of p6F prior to binding. Our results further demonstrate the sensitivity of the TCR recognition mechanisms for the pMHC complex structure and dynamics, providing further tools to investigate and modulate TCR binding and immunogenicity via APLs.

## Materials and Methods

### Molecular Dynamics Simulations

Molecular dynamics simulations of gp33 variants alone in solution, in complex with H-2D^b^, and of each H-2D^b^/peptide complex bound to the TCR P14 were performed using GROMACS 2018^54^ and PLUMED2.^55^ The wild-type peptide gp33 (KAVYNFATM) and all other variants were built as linear peptides using Pymol.^56^ Simulations of both H-2D^b^/pep and P14/H-2D^b^/pep complexes were setup based on the crystal structures of H-2D^b^/gp33, H-2D^b^/V3P, P14/H-2D^b^/gp33, and P14/H-2D^b^/V3P, with PDB codes 1S7U,^35^ 4NSK, 5TJE, and 5TIL,^25^ respectively. In the case of TCR P14, only the variable domains were retained for the simulation. All missing amino acids were modeled using Modeller.^57^ The d-amino acid variant p6f of the peptide gp33 was modeled from the corresponding wild-type structure using Pymol. All simulations were performed using the Amber99SB-disp^58^ with the TIP4P-D water model,^59^ a force field recently developed and extensively tested to work both with ordered and disordered proteins. Each system was solvated into a dodecahedron box with a salt concentration of 15 mM of NaCl. Temperature and pressure were controlled using the velocity rescale^60^ and Parrinello–Rahman^61^ algorithms, respectively. Short-range Coulomb and van der Waals interactions were cut off at 0.9 nm and long-range Coulomb interactions were calculated using the particle mesh Ewald method.^62^ All systems were equilibrated using a preliminary energy minimization step, followed by a 200 ps long *NVT* simulation with the proteins’ atoms restrained to their minimized positions, eventually followed by a 1 ns NPT simulation using the Berendsen’s barostat.^63^ Subsequently, all systems were simulated for 500 ns at a temperature of 309 K and a pressure of 1 atm. Peptides’ simulations were evolved with a time step of 2 fs constraining only bonded hydrogens. For H-2D^b^/pep and P14/H-2D^b^/pep simulations, the hydrogen mass repartitioning scheme^64^ was used to reduce the computational cost. The mass of heavy atoms was repartitioned into the bonded hydrogen atoms using the *heavyh* flag in the pdb2gmx tool. The LINCS algorithm^65^ was used to constrain all bonds, eventually allowing to use a time step of 5 fs.

### Metadynamics Simulations

The conformational space accessible to the gp33 variants in solution, in complex with H-2D^b^ and bound to the TCR P14, was sampled making using of Parallel Bias Metadynamics (PBMetaD) simulations.^36,66^ This metadynamics approach allows enhancing the sampling of multiple one-dimensional (1d) collective variables (CVs), providing a solution to the issue of choosing only a few CVs to describe all the slow motions of the system. All the simulations were performed adopting the multiple-walker scheme,^67^ simulating seven replicas (or walkers) for the peptide in solution and four replicas for the H-2D^b^/pep and P14/H-2D^b^/pep complexes. Each replica was evolved for 200, 500, and 200 ns for the peptides in solution, as well as for the H-2D^b^/pep and P14/H-2D^b^/pep complexes, respectively. For peptides in solution, the selected CVs were the torsional angles phi, psi, χ_1_, and χ_2_ of each residue, if defined. For the H-2D^b^/pep and P14/H-2D^b^/pep complexes, phi angles of peptide residues 3–7, psi angle of residue 6, and χ_1_, χ_2_ of residues 4 and 6 were employed. Gaussians were deposited every 1 ps with an initial height of 0.5 kJ/mol using a bias factor of 10. For all the CVs, the width of the Gaussians was set to 0.1 rad. Each simulation was analyzed by creating a concatenated trajectory and reweighting each frame by using the final metadynamics bias potential, assuming a constant bias during the entire course of the simulation.^68^

### Peptide Synthesis

Peptides were synthesized on preloaded Wang resin (0.43 loading) using a CEM Liberty peptide synthesizer on a 0.1 mmol scale following standard protocols.^69,70^ The amino acid concentration was equal to 0.2 M in DMF. DIC and Oxyma were used as coupling reagents (0.1 M in DMF), while for the deprotection 20% Piperidine in DMF was used. Couplings were performed at 75 °C using 170 W for 15 s and then at 90 °C using 40 W for 110 s. Deprotection was performed at 75 °C using 155 W for 15 s and then at 90 °C using 50 W for 50 s. Double couplings were performed for alanine, proline, and tyrosine residues. The cleavage was then performed using a total of 3 mL of cleavage cocktail for each peptide (trifluoroacetic acid/thioanisole/3,6-dioxa-1,8-octanedithiol; 92:5:3) for 180 min. After cleavage, the peptides were precipitated and washed using ice-cold diethyl ether. All produced peptides were purified using an ADAMAS C-18 column from Sepachrom (10 μm, 250 × 21.2 mm) by RP-HPLC using a gradient elution of 15–60% solvent B (solvent A: water/trifluoroacetic acid 100:0.1; solvent B: acetonitrile/trifluoroacetic acid 100:0.1) over 40 min at a flow rate of 20 mL/min. The purified peptides were lyophilized and stored at 0 °C.

### Refolding and Isolation of pMHC Complexes

The murine H-2D^b^ heavy chain and mouse β2m (mβ2m) were expressed individually as inclusion bodies using BL21 (DE3) *E. coli* strain, following previously published protocols.^71−74^ gp33, V3P, F6f, and V3P_F6f peptides were synthetized and used to refold pMHC, yielding H-2D^b^/gp33, H-2D^b^/V3P, H-2D^b^/F6f, and H-2D^b^/V3P_F6f, respectively. H-2D^b^/peptide complexes were obtained through dilution.^75^ Briefly, peptide (10 μM) and mβ2m (2 μM) were incubated in the refolding buffer (100 mM Tris pH 8, 450 mM l-arginine, 5 mM l-glutathione reduced, 0.5 mM l-glutathione oxidized, 2 mM EDTA, and 0.5 mM AEBSF) at 4 °C under stirring for 30 min. The unfolded H-2D^b^ heavy chain, solubilized in 6 M guanidinium hydrochloride, was thereafter added to a final concentration of 1 μM. The refolding was completed after 72 h at 4 °C under stirring. The solution was concentrated using Stirred Ultrafiltration Cell (Millipore) and Amicon Ultra-15 Centrifugal Filters (EMD Millipore). Finally, all pMHC complexes were purified by size exclusion chromatography using a HiLoad 16/600 Superdex 200 pg column (GE Healthcare), preliminarily equilibrated in 20 mM Tris–HCl pH 7.4.

### Circular Dichroism Analysis of pMHC Complex Thermal Stability

Thermal unfolding experiments were performed in the far-UV region on a J-810 spectropolarimeter (JASCO Corp., Tokyo, Japan) equipped with a Peltier system for temperature control. Measurements were performed in 20 mM Tris–HCl pH 7.4 using a 0.2 mg/mL protein concentration. The temperature ramp measurements were recorded from 20 to 95 °C (temperature slope 60 °C/h) in a 0.1 cm path length cuvette and monitored at a 218 nm wavelength. The T_m_ values were calculated as the first-derivative minimum of the traces. Curves and T_m_ values are an average of at least three measurements from at least two independent refolding assays per pMHC. Spectra were analyzed using GraphPad Prism 5 (La Jolla, USA).

### Crystallization of H-2D^b^/F6f and H-2D^b^/V3P_F6f

Crystallization experiments were performed at 293 K using the sitting drop vapor diffusion method by mixing an equal amount of 7 mg/mL of each pMHC complex in 20 mM Tris–HCl pH 7.4 and reservoir solution. Best diffracting crystals were obtained under the conditions of 1.7 M ammonium sulfate, 100 mM Tris–HCl, and pH 8.3. Crystals were cryoprotected with 20% glycerol and flash-frozen in liquid nitrogen. X-ray diffraction data were collected at the beam line XRD-2 at Elettra (Trieste, Italy).

### Determination of the Crystal Structures of H-2D^b^/F6f and H-2D^b^/V3P_F6f

Crystals of H-2D^b^/F6f and H-2D^b^/V3P_F6f diffracted to 2.6 and 2.4 Å, respectively. Both crystals belong to the space groups C2_1_. Data collection statistics are presented in Table 1. Diffraction data were processed using Staraniso,^76^ and intensities were merged with AIMLESS. The crystal structures of H-2D^b^/F6f and H-2D^b^/V3P_F6f were determined by molecular replacement using PhaserMR and the previously determined crystal structure of H-2D^b^/gp33 (PDB 1S7U)^35^ and H-2D^b^/V3P (PDB 4NSK)^25^ as search models, respectively. In both cases, two pMHCs were present in the asymmetric unit. The molecular models were preliminary subjected to a rigid-body refinement, followed by a restrained refinement using phenix.phaser.^77^ Manual model building was thereafter carried out using COOT.^78^

### SPR Binding Affinity Analysis

All measurements were performed on a BIAcore 2000 (GE Healthcare, USA) at 25 °C. Soluble TCR P14 (20 μg/mL) was non-covalently coupled to the anti-C_β_ antibody H57-597. 8000 RUs of H57-957 were coupled to a CM5-chip, resulting in 3000 RUs immobilized P14. A control surface without antibody was used as reference. Concentration series of pMHCs were injected over the chip. The surface was regenerated with 40 μL 0.1 M Glycine-HCl, 500 mM NaCl, and pH 2.5. Unspecific binding was corrected for by subtracting responses from reference flow cells. Data were analyzed with BIAevaluation 2000 (BIAcore AB, Uppsala, Sweden). *K*_D_ values were obtained from steady-state fitting of equilibrium binding curves from at least two independent measurements.

## Supporting Information Available

Principal component analysis; additional free energy surfaces; and distribution of distances for specific couple of residues (PDF)

## Accession Codes

The atomic coordinates and related experimental data for H-2D^b^/V3P-_F6f and H-2D^b^/F6f system studies in this work are deposited in the Protein Data Bank with accession code 7P0A and 7P0T.

## Abbreviations

gp33: wild-type, KAVYNFATM
V3P: KA**P**YNFATM
Y4F: KAV**F**NFATM
V3P_Y4F: KA**PF**NFATM
F6f: KAVYN**f**ATM
V3P_F6f: KA**P**YN**f**ATM
lower-case **f**: d-phenylalanine
The p[Position][ResName] nomenclature: the residue [ResName] in peptide position [Position].
MHC: major histocompatibility complex
TCR: T cell receptor
CTL: cytotoxic CD8+ T lymphocyte
LCMV: lymphocytic choriomeningitis virus
TAA: tumor-associated antigen
APL: altered peptide ligand
pMHC or H-2Db/pep: MHC peptide complex
P14/H-2Db/pep: H-2Db/pep in complex with the TCR P14
MD: molecular dynamics simulations
FES: free energy surface
RMSF: root mean square fluctuations
rmsd: root mean square deviations
