# Supplementary material for: l- to d-Amino Acid Substitution in the Immunodominant LCMV-Derived Epitope gp33 Highlights the Sensitivity of the TCR Recognition Mechanism for the MHC/Peptide Structure and Dynamics
Source: ACS Omega. 2022 Mar 7;7(11):9622–35. doi: 10.1021/acsomega.1c06964 (PMC8945122; doi:10.1021/acsomega.1c06964)
Supplement: Supplementary file 1 — ao1c06964_si_001.pdf [file ao1c06964_si_001.pdf]

Supplementary Information

An L- to D-amino acid substitution in the immunodominant LCMV-derived epitope gp33 highlights the sensitivity of the TCR recognition mechanism for the MHC/peptide structure and dynamics

Federico Ballabio[1,‡], Luca Broggini[1,2,‡], Cristina Paissoni[1,‡], Xiao Han[3,‡], Kaliroi Peqini[4], Benedetta Maria Sala[3], Renhua Sun[3], Tatyana Sandalova[3], Alberto Barbiroli[5], Adnane Achour[3,], Sara Pellegrino[4,*], Stefano Ricagno[1,2,*], Carlo Camilloni[1,*]*

[1]Dipartimento di Bioscienze, Università degli Studi di Milano, Milano, Italy

[2] Institute of Molecular and Translational Cardiology, IRCCS Policlinico San Donato, San Donato Milanese, Italy

[3]Science for Life Laboratory, Department of Medicine, Karolinska Institute, & Division of Infectious Diseases, Karolinska University Hospital, Stockholm, Sweden

[4]DISFARM, Dipartimento di Scienze Farmaceutiche, Sezione Chimica Generale e Organica, Università degli Studi di Milano, Milano, Italy

[5]Dipartimento di Scienze per gli Alimenti, la Nutrizione e l'Ambiente, Università degli Studi di Milano, Milano, Italy

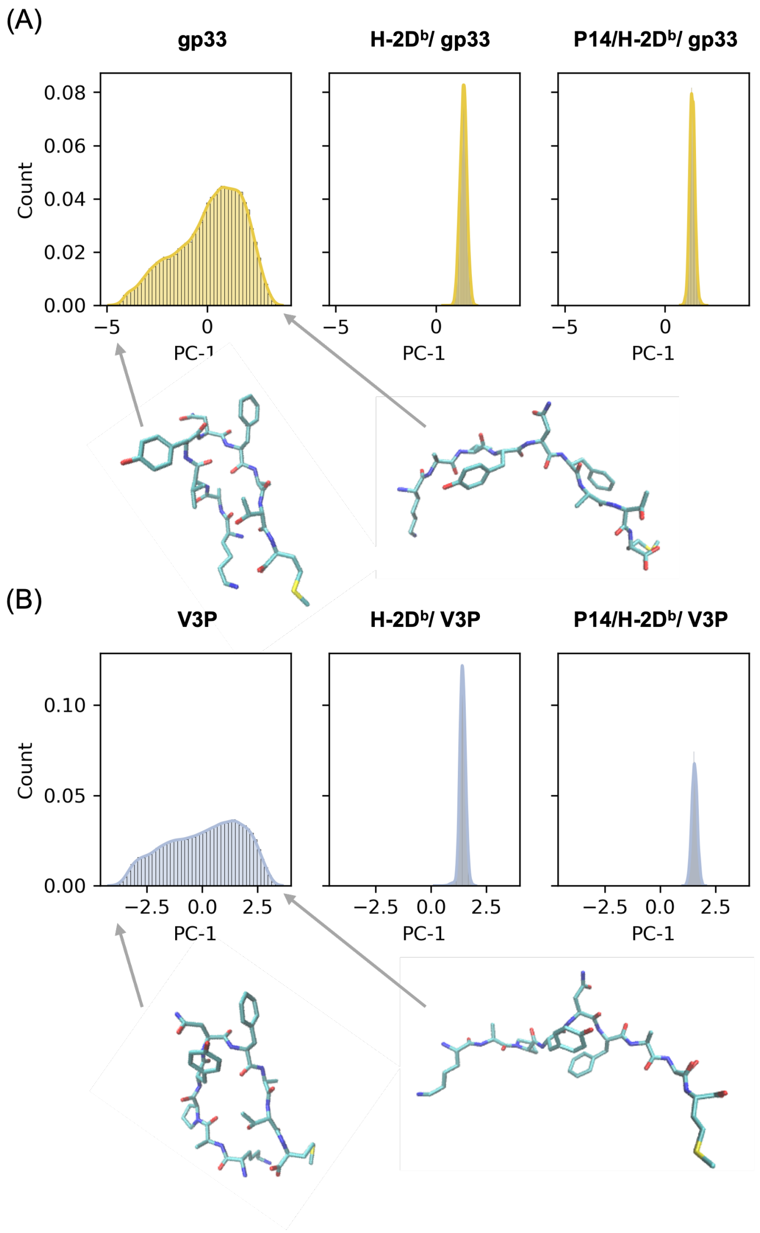

Figure S1. Principal Component Analysis of the gp33 and V3P simulations. Principal Component Analysis (PCA) was performed on the simulations of gp33 (top, A) or V3P (bottom, B) peptides free in solution. In the plots are shown the projections of pep, H-2D^{b}/pep and P14/H-2D^{b}/pep simulations on the first eigenvector found from the PCA of the free peptide. The frames corresponding to the lowest and highest values of the first principal component are displayed in licorice. For both gp33 and V3P, the first component is related to the compactness of the peptide;

loading of the peptide on MHC (H-2D^b/pep), as well as the binding of this complex to TCR (P14/H-2D^b/pep), result in a restriction of the peptide conformational freedom which is then restrained in an extended conformation.

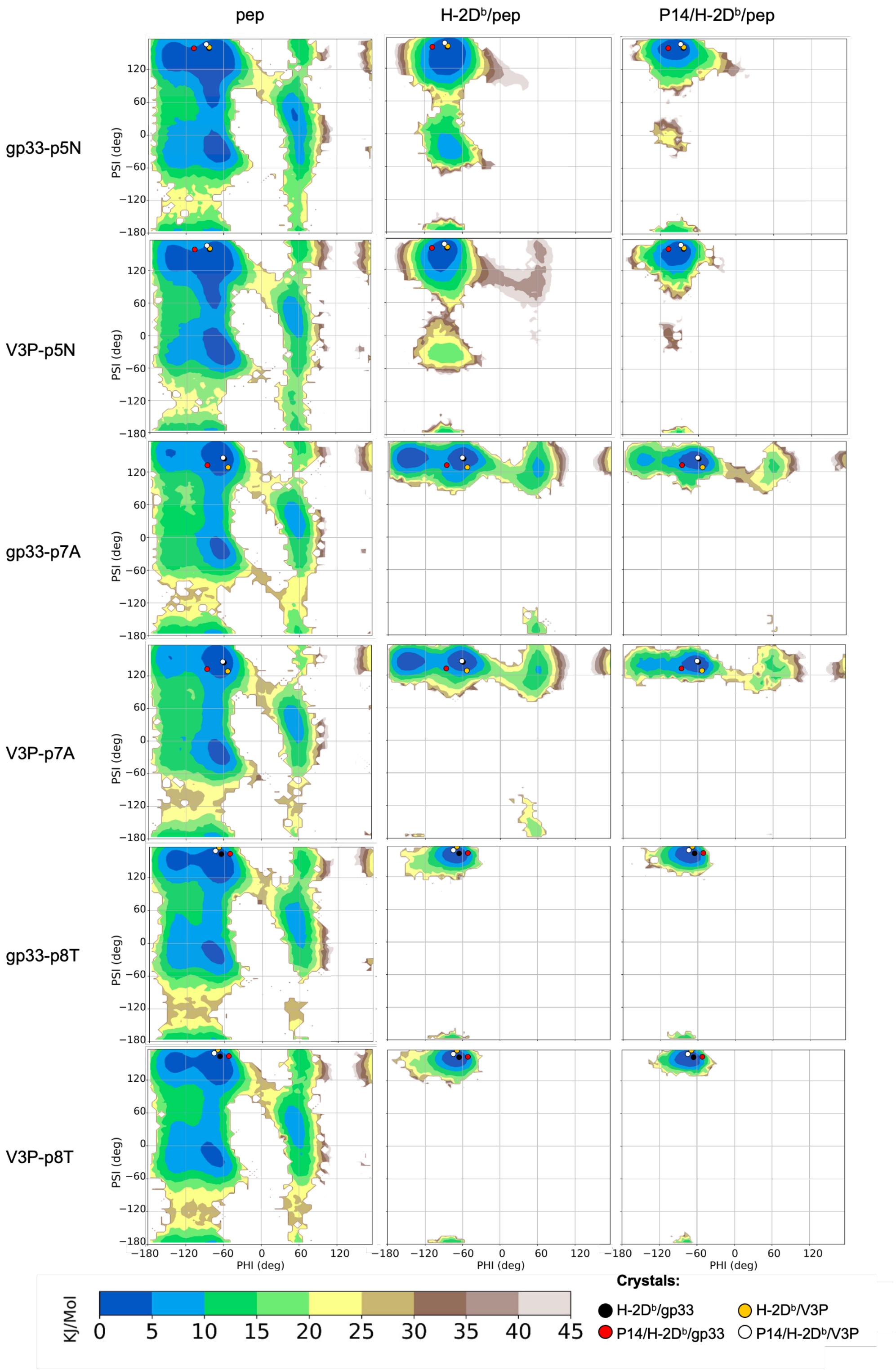

Figure S2. Ramachandran-like free energies indicate that the p3P modification does not reduce the conformational freedom of peptide residues p5N, p7A and p8T. The 2d-free

energies surfaces are presented, as a function of the phi and psi dihedral angles, for residues p5N (top panels), p7A (middle panels) and p8T (bottom panels). The free energies are reported for both gp33 and V3P , for the simulations of the peptides alone (left), H-2D^b/pep complexes (middle) and P14/H-2D^b/pep ternary complexes (right). In all the plots, the coordinates for each peptide residue derived from the previously determined crystal structures of pMHC and TCR/pMHC comple are plotted as coloured dots.

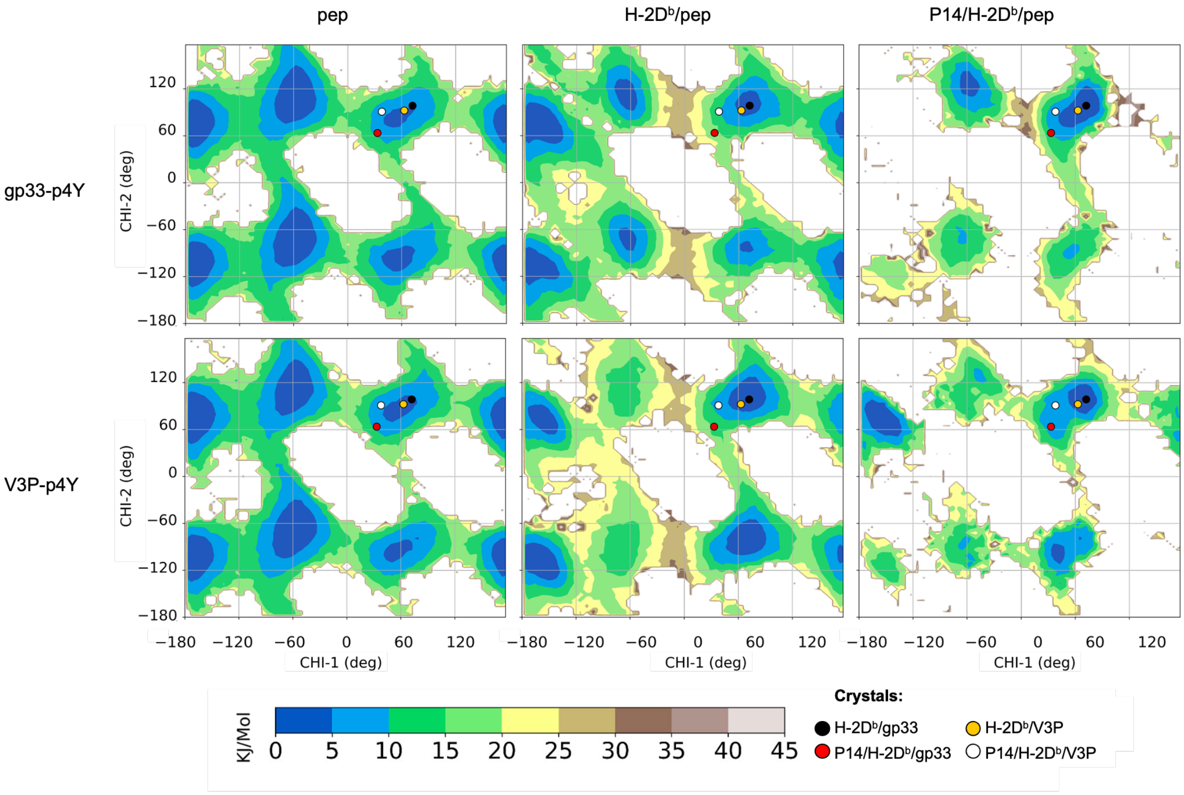

Figure S3. The p3P modification in V3P reduces the number of available conformational minima for the sidechain of residue p4Y when bound to H-2D^b as monitored by side chain χ1- χ2 free energies. The free energies are reported for both the gp33 and the V3P peptides, for the simulations of the peptide alone (left), H-2D^b/pep complexes (middle) and P14/H-2D^b/pep ternary complexes (right). In all the plots, the same coordinates derived from the previously determined crystal structures of pMHC and TCR/pMHC complexes are plotted as coloured dots.

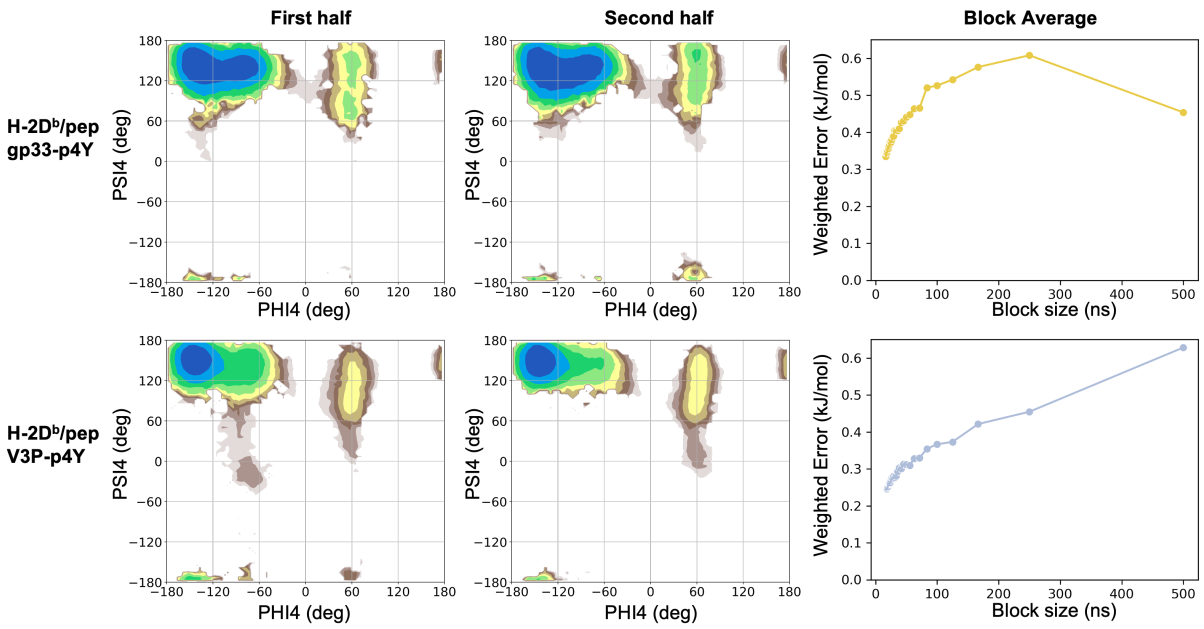

Figure S4. The 2d-free energies are well converged. 2d-free energies, as a function of p4Y phi and psi dihedral angles, for both the gp33 (top) and the V3P (bottom) peptides, for the simulations of the H-2D^b/pep complexes. The 2d-free energies were built considering separatingly the first half (left panel) and the second half (middle panel) of the simulations: free energies from each half are comparable and reproduce the observed differences among the gp33 and V3P peptides indicating that they are well converged. In the right panel the block average analysis is shown. The simulations have been divided in blocks of different lengths, up to 500 ns (i.e., the length of each replica), and the weighted sum of free-energy errors was computed for each length.

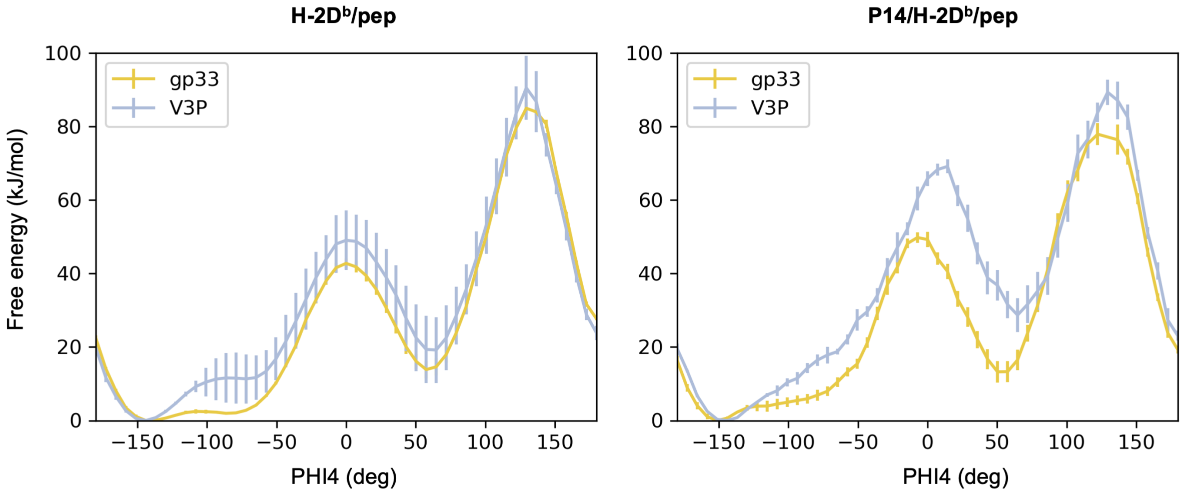

Figure S5. Comparison of 1d-free energy profiles for the p4Y phi dihedral angle. The free energies are shown for gp33 (yellow) and V3P (blue) peptides, both in H-2D^b/pep (left) and P14/H-2D^b/pep (right) complexes. The error bars indicate the errors computed from block average analysis with 500 ns block size.

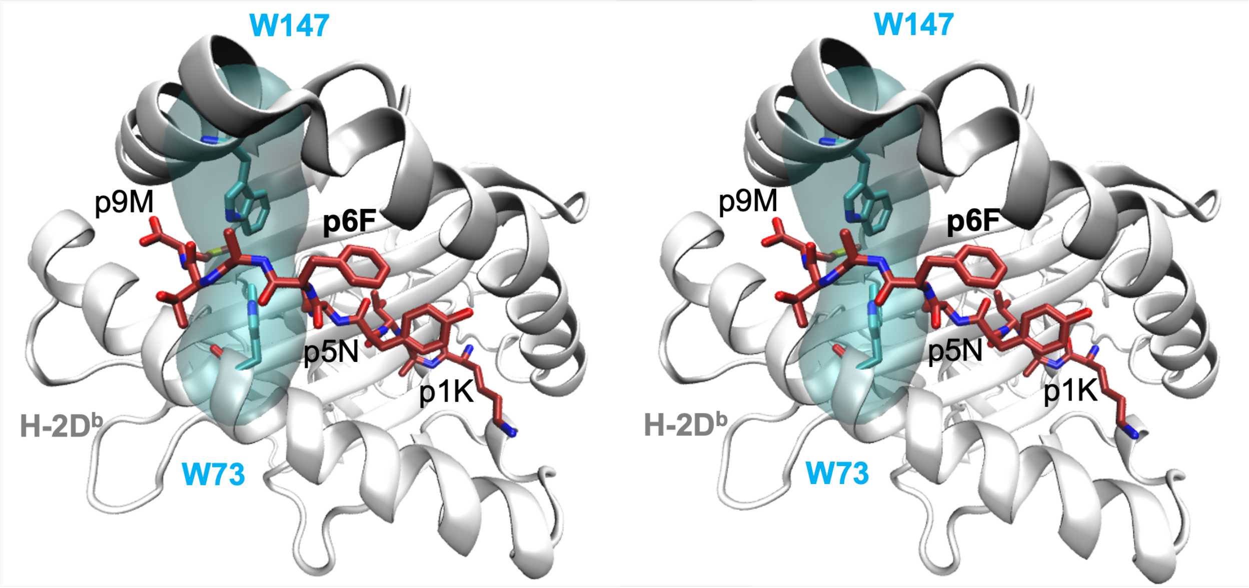

Figure S6. Tryptophan residues W73 and W147 create a bulge in the H-2D^b binding groove, resulting in a unfavorable conformation for peptide residue p6. Stereoview of peptide gp33 bound to H-2D^b. The backbone of the H-2D^b binding pocket is displayed in white. The peptide gp33 is represented in red sticks, with oxygen and nitrogen atoms in deep red and blue, respectively. The two H-2D^b tryptophan residues W73 and W147, highlighted with cyan sticks and surface, form a bulge underneath the presented peptide, stretching the section p6-p8 between the two main anchor positions p5N and p9M.

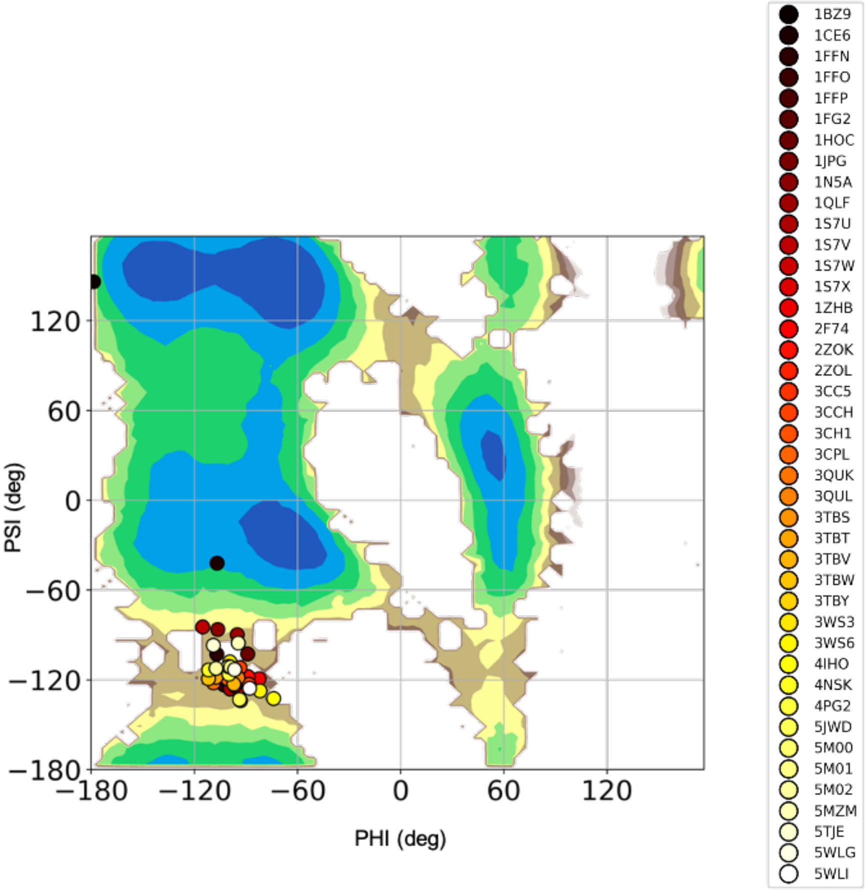

Figure S7. Phi and psi dihedral angles of peptide residue p6 from available H-2D^b/pep crystal structures reveal the highly strained conformation of residue p6. The phi and psi dihedral angles of peptide residue p6 were computed and plotted with dots for 42 crystal structures of H-2D^b in complex with a nonameric peptide (PDB codes and relative colors are listed to the right). To provide a reference, it is plotted the 2d-free energy surface, as a function of the phi and psi dihedral angles of residues p6F, for gp33 peptide free in solution according to our simulations, all but two structures (i.e. PDBs 1CE6 and 1BZ9) correspond to high free energy configurations for the amino acid in solution (cf. the color bar in Figure S2).

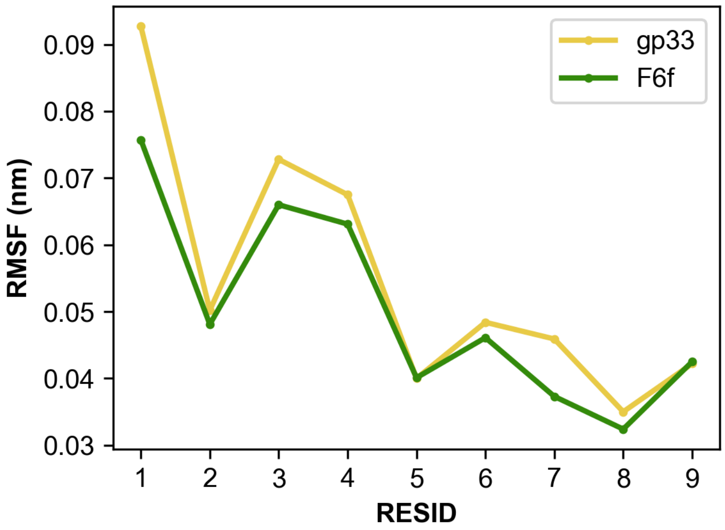

Figure S8. The L- to D- modification in F6f does not affect significantly peptide flexibility within H-2D^b. Per-residue backbone Root Mean Square Fluctuations (RMSF) of peptides gp33 and F6f from H-2D^b/pep MD simulations do not indicate any significant differences.

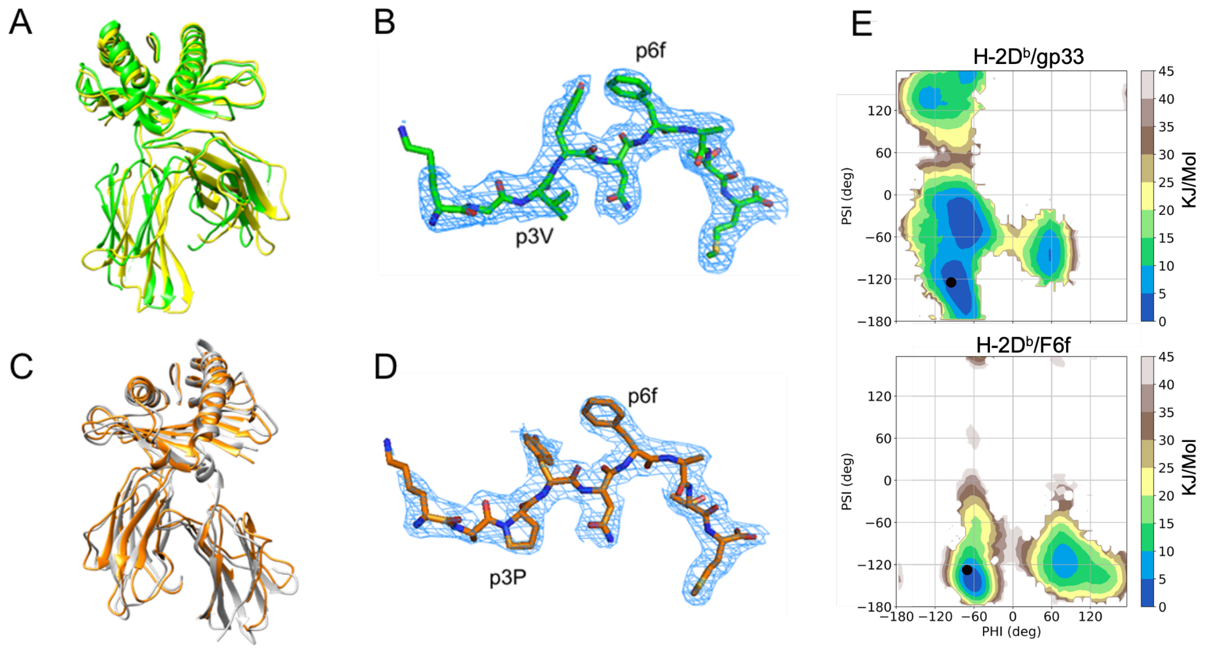

Figure S9. Crystal structures of H-2D^b in complex with F6f and V3P_F6f. A and C) The overall folds of H-2D^b/F6f (green) and H-2D^b/V3P_F6f (orange) are very similar to H-2D^b/gp33 (yellow) and H-2D^b/V3P (grey). The conformations of the L- to D-modified peptides are similar to their counterparts (rmsd values of 0.3Å for H-2D^b/ F6f compared to H-2D^b/gp33, and 0.5Å for H-2D^b/V3P_F6f compared to H-2D^b/V3P. **B and D)** Side views of the peptides F6f and V3P_F6f, with the annotated modified side chain. The 2Fo-Fc electron density maps, colored in blue, allow for unambiguous positioning of all peptide residues. **E)** The 2d-free energies surface from MD simulation, as a function of residue p6 phi and psi dihedral angles, are presented for H-2D^b/gp33 (top) and H-2D^b/F6f (bottom). The coordinates for peptide residue p6 of the corresponding crystal structures in that space are plotted as black dots.

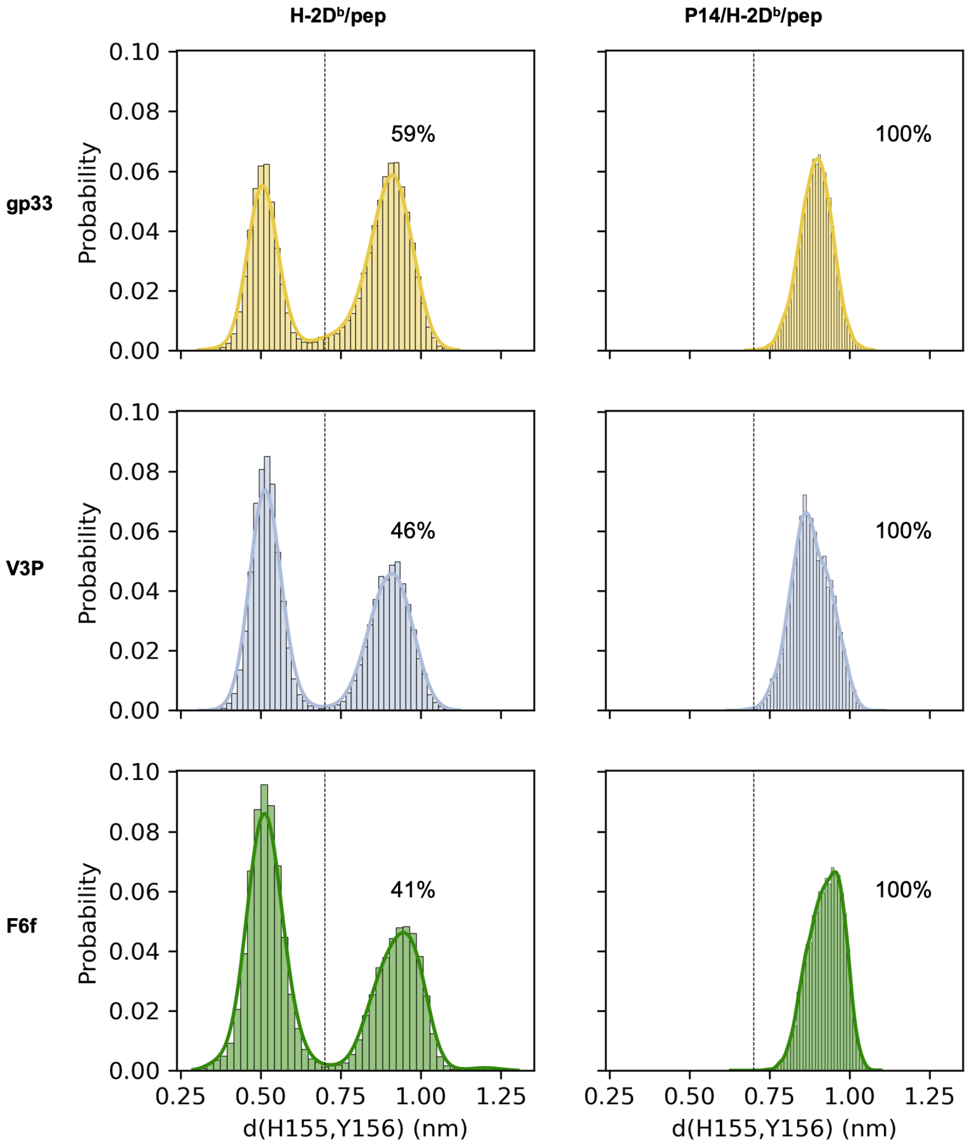

Figure S10. Position of H-2D^b residue H155. The inward or outward orientation of H155 is monitored by measuring the distance between the atoms H155:NE2 and Y156:CZ. Distances smaller than 0.7 nm are indicative of inward orientation and vice-versa. In the plots are represented the probability distributions of this distance for gp33, V3P and F6f peptides in either H-2D^b/pep (left) or P14/H-2D^b/pep (right panel) simulations. For each plot the percentage of frames in which H155 adopts the ideal outward orientation is indicated.

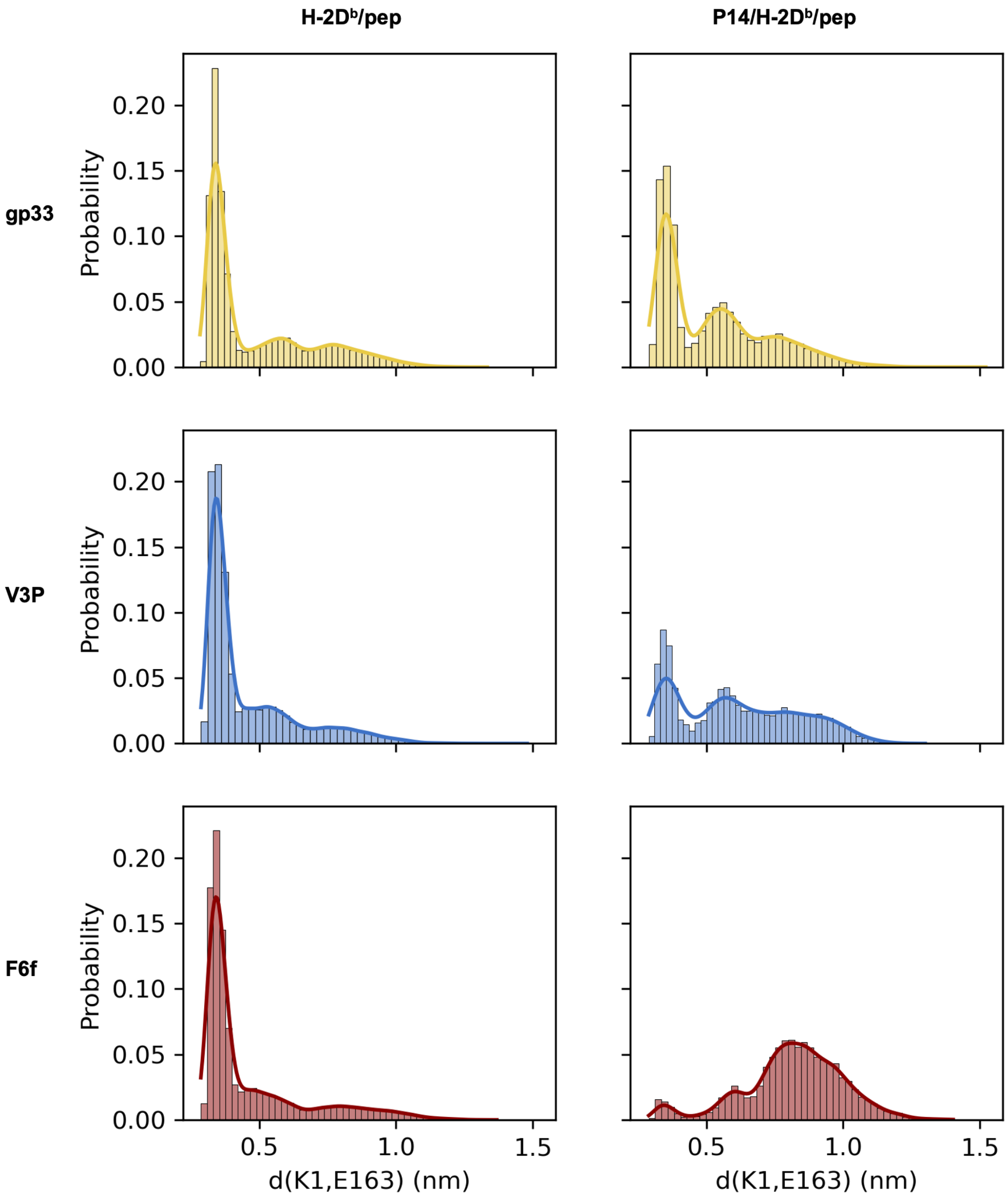

Figure S11. Position of H-2D^b residue E163 with respect to peptide residue p1K. The distance between the atoms E163:CD and p1K:NZ is monitored. In the plots are represented the probability distributions of this distance for gp33, V3P and F6f peptides in either H-2D^b/pep (left) or P14/H-2D^b/pep (right panel) simulations.

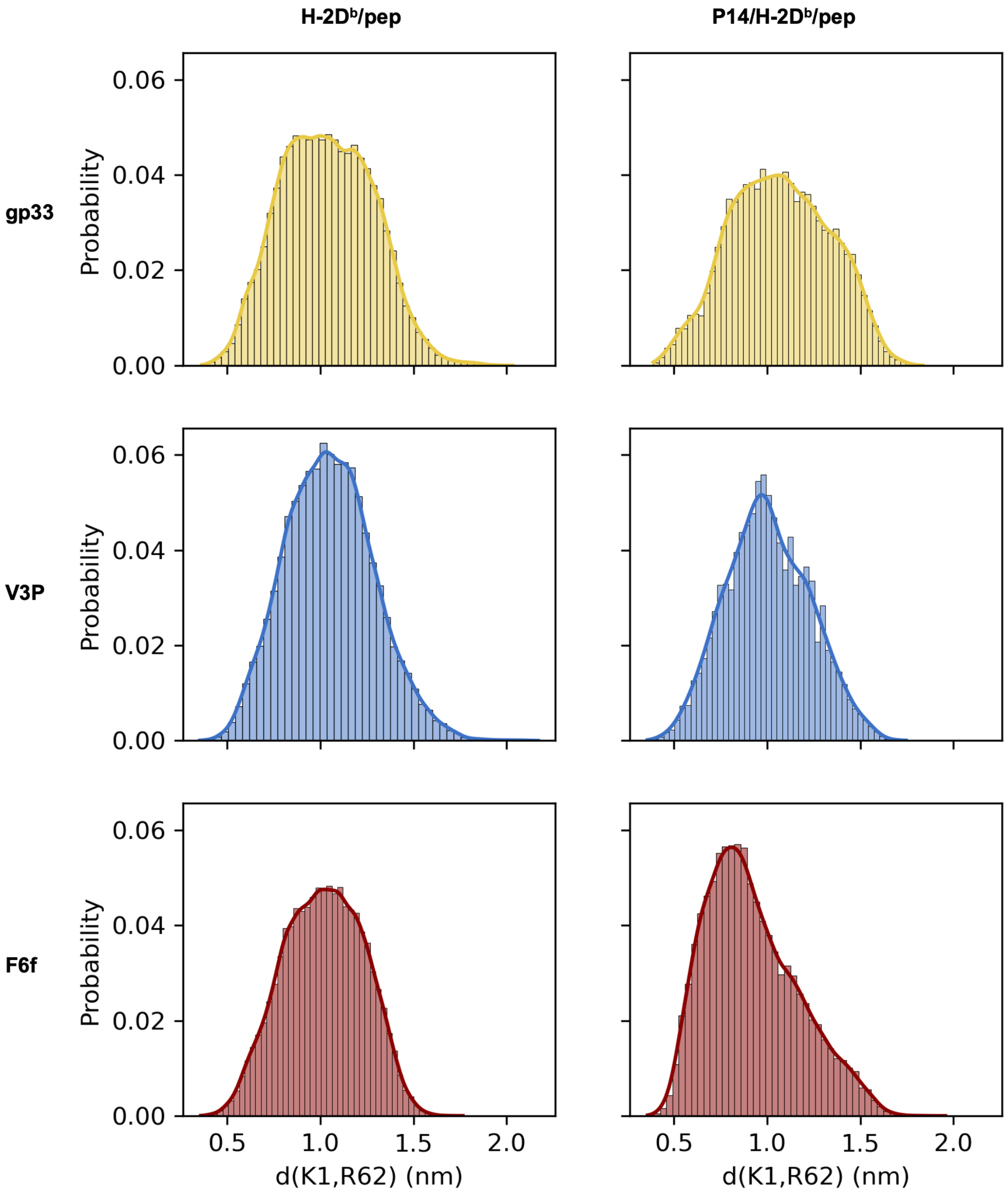

Figure S12. Position of H-2D^b residue R62 with respect to peptide residue p1K. The distance between the atoms R62:CZ and p1K:NZ is monitored. In the plots are represented the probability distributions of this distance for gp33, V3P and F6f peptides in either H-2D^b/pep (left) or P14/H-2D^b/pep (right panel) simulations.
